# Factorization of the Green’s function of high-frequency acoustic half-space impedance problems

**DOI:** 10.1007/s00033-026-02782-0

**Published:** 2026-05-06

**Authors:** C. Lin, J. M. Melenk, S. Sauter

**Affiliations:** 1 Institut für Mathematik, Universität Zürich, Winterthurerstrasse 190, CH-8057 Zürich, Switzerland; 2 Institut für Analysis und Scientific Computing, Technische Universität Wien, Wiedner Hauptstrasse 8-10, A-1040 Wien, Austria

**Keywords:** Acoustic scattering, Impedance half-space, Green’s function, Geometric optics, Bessel functions, 33C10, 35J08, 41A10

## Abstract

We show that the acoustic Green’s function for a half-space impedance problem in arbitrary spatial dimension *d* can be written as a sum of three terms, each of which is the product of an exponential function with the *eikonal* in the argument and a *slowly varying* function. We introduce the notion of families of slowly varying functions to formulate this statement as a theorem and present its proof.

## Introduction

Wave phenomena in half-space domains have many important applications such as the modelling of noise propagation over flat ground, the detection of inclusions in homogeneous soil under a plane crust, as well as the design of noise barriers positioned over a large flat boundary of an ambient domain modelled by a half-space. For *high-frequency* scattering problems, a mathematical formulation by a Helmholtz equation in the frequency domain is appealing (see e.g. [10, 12, 22]). The ultimate goal is the reliable numerical simulation of such phenomena, and the boundary element method (see, e.g. [33]) is a well-established and popular numerical discretization method for acoustic problems in unbounded domains. Typically this involves the explicit knowledge of the full-space Green’s function of the underlying differential operators along with an analysis of its specific properties. While for half-space problems with sound-hard or sound-soft boundary conditions the Green’s function is available by the classical method of images, it is not explicitly available for the physically more relevant case of impedance boundary conditions; typically, it is expressed by oscillatory Fourier-type integrals, Hankel transforms, and Sommerfeld-type integrals (see, e.g. [11, (13)], [15, 16, 17, 19, 21, 28, 31, (22)]). While standard methods from asymptotic analysis such as the method of stationary phase allow for detecting the lowest order term in an asymptotic expansion, it is a fairly open problem to obtain uniform higher-order asymptotic expansions for this Green’s function as well as estimates of the remainder and structural insight in this function.

It is the main goal of this paper to develop an analysis of the acoustic half-space Green’s function for impedance boundary conditions tailored to the development of fast numerical solution methods of the corresponding boundary integral equations; for the free-space Green’s function, we refer in this context to [2–5, 18, 26, 32]. We emphasize that the focus of the present paper is on the analysis of the half-space Green’s function, while the development of fast numerical solution methods is a topic of future research. Our starting point is a new representation of the half-space acoustic impedance Green’s function in arbitrary spatial dimension $d\in \left\{ 1,2,3,\ldots \right\}$ that is derived in the companion paper [23]. In contrast to the representations via oscillatory integrals, the integrand in the new integral representation is non-oscillatory and defines a function that is non-oscillatory in a sense which will be made precise in this paper.

The analysis in our paper is based on this new representation and makes the following statement rigorous:

$$\begin{aligned}&{\text {The acoustic Green's function for a half-space impedance problem can be written as the}}\nonumber \\&{\text {sum of (three) terms, each of which is the product of an oscillatory exponential}}\\&{\text {function and a slowly varying function.}} \qquad \qquad \qquad ({\textrm{S}}) \end{aligned}$$

This is in the spirit of the theory of geometric optics and ray theory; standard references include [1, Chap. 2], [7, 8, Chap. 3] where scattering problems are analysed using (the sum of) products of the exponential function with the eikonal in the argument and a slowly varying function. Here our focus is the development of a rigorous analysis of the slowly varying function for the specific half-space problem.

Our analysis has immediate implication for its approximation: the slowly varying part can be approximated by polynomials with exponential convergence, and the oscillatory exponential factor can be treated by directional hierarchical ($\mathcal{D}\mathcal{H}^{2}$) matrices (see [5, 6, 18]) or by a butterfly algorithm [9].

The paper is structured as follows: In Sect. 2, we formulate the governing equations for the acoustic half-space Green’s function and briefly recall the representation from [23]. In Sect. 3, we introduce the notion of slowly varying functions and explain/analyse the approximation by tensor Chebyshev interpolation. This is the prerequisite for the analysis of the Green’s function in Sect. 4 so that statement (S) can be made rigorous in Theorem 4.2. Section 4 starts with the derivation of a majorant of the modified Bessel function of the second kind, which is immanent in the representation of the Green’s function (Sect. 4.1), and continues with the investigation of holomorphic norm extensions (Sect. 4.2), and the final step provides an analysis of a coordinate transform, which is involved in the integral representation of the impedance part of the half-space Green’s function (see Sect. 5).

## The Green’s function for the acoustic half-space problem with impedance boundary conditions

Let the upper/lower half-space in $\mathbb {R}^{d}$, $d\in \left\{ 1,2,\ldots \right\}$, and its boundary be denoted by:

$$\begin{aligned} H_{+}&:=\left\{ \textbf{x}=\left( x_{j}\right) _{j=1}^{d}\in \mathbb {R}^{d}\mid x_{d}>0\right\} ,\\ H_{-}&:=\left\{ \textbf{x}=\left( x_{j}\right) _{j=1}^{d}\in \mathbb {R}^{d}\mid x_{d}<0\right\} ,\\ H_{0}&:=\partial H_{+}:=\left\{ \textbf{x}=\left( x_{j}\right) _{j=1}^{d}\in \mathbb {R}^{d}\mid x_{d}=0\right\} \end{aligned}$$

with outward normal vector $\textbf{n}=\left( 0,\ldots ,0,-1\right) ^{T}$. Let

$$ \overset{\bullet }{\mathbb {C}}_{\ge 0}:=\left\{ \zeta \in \mathbb {C} \mid \operatorname {Re}\zeta \ge 0\right\} \backslash \left\{ 0\right\} \quad {\text {and}}\quad \mathbb {C}_{>0}:=\left\{ \zeta \in \mathbb {C} \mid \operatorname {Re}\zeta >0\right\} . $$

We consider the problem to find the Green’s function $G:H_{+}\times H_{+}\rightarrow \mathbb {C}$ for the acoustic half-plane problem with impedance boundary conditions:

2.1 $$\begin{aligned} \begin{array}{rll} -\Delta _{\textbf{x}}G\left( \textbf{x},\textbf{y}\right) +s^{2}G\left( \textbf{x},\textbf{y}\right) &  =\delta _{0}\left( \textbf{x}-\textbf{y} \right) &  {\text {for}}\left( \textbf{x},\textbf{y}\right) \in H_{+}\times H_{+},\\ \frac{\partial }{\partial \textbf{n}_{\textbf{x}}}G\left( \textbf{x} ,\textbf{y}\right) +s\beta G\left( \textbf{x},\textbf{y}\right) &  =0 &  {\text {for}}\left( \textbf{x},\textbf{y}\right) \in H_{0}\times H_{+}\\ G\left( r \boldsymbol{\zeta } ,\textbf{y}\right) &  \overset{r\rightarrow +\infty }{\rightarrow }0 &  {\text {for}}\left( {\boldsymbol{\zeta }} ,\textbf{y}\right) \in H_{+}\times H_{+} \end{array} \end{aligned}$$

for some β>0 and frequency $s\in \mathbb {C}_{>0}$. The index $\textbf{x}$ in the differential operators indicates that differentiation is done with respect to the variable $\textbf{x}$.

### Remark 2.1

Problem (2.1) is formulated for $s\in \mathbb {C}_{>0}$. The Green’s function $G=G_{s}$ depends on *s* and for $\operatorname {Re}s>0$ it is assumed to decay for $\textbf{x}=r {\boldsymbol{\zeta }}$ as $r\rightarrow +\infty$ for any fixed direction ${\boldsymbol{\zeta }} \in H_{+}$. Problem (2.1) for the case $s\in \operatorname {i} \mathbb {R}\backslash \left\{ 0\right\}$ is considered as the limit from the positive complex half-plane $\mathbb {C}_{>0}$:

$$ G_{s}=\lim _{\begin{array}{c} \zeta \rightarrow s\\ \zeta \in \mathbb {C}_{>0} \end{array}}G_{\zeta }{\text {.}} $$

In the following, we focus our attention on spatial dimensions d≥2 and general impedance parameter β>0. In [23], fully explicit representations of the Green’s function are presented for d=1 and for d>1 with β=1. Next we recall the new integral representation given in [23].

The representation of the Green’s function as the solution of (2.1) requires some preparations. Let $K_{\nu }$ denote the Macdonald function (modified Bessel function of the second kind and order ν, see, e.g. [14, §10.25], [24]). The dependence on the spatial dimension *d* will be expressed via the parameter

$$ \nu :=\left( d-3\right) /2. $$

We introduce the function

2.2 $$\begin{aligned} g_{\nu }\left( r\right) :=\frac{1}{\left( 2\pi \right) ^{\nu +3/2}}\left( \frac{s}{r}\right) ^{\nu +1/2}K_{\nu +1/2}\left( sr\right) \end{aligned}$$

and note that $g_{\nu }\left( \left\| \textbf{x}-\textbf{y}\right\| \right)$ is the full space Green’s function for the Helmholtz operator (see [25, Thm. 4.4] and [8, (6), (12)] in combination with the connecting formula [14, §10.27.8]). For $\mathbf {y=} \left( y_{j}\right) _{j=1}^{d}\in H_{+}$, we introduce the reflection operator $\textbf{Ry}:=\left( \textbf{y}^{\prime },-y_{d}\right)$, where $\textbf{y}^{\prime }=\left( y_{j}\right) _{j=1}^{d-1}$. Let

$$ \mathbb {Z}^{\operatorname {half}}:=\left\{ -\frac{1}{2},0,\frac{1}{2},1,\ldots \right\} \quad {\text {and}}\quad \mathbb {Z}_{\ge \mu }^{\operatorname {half}}:=\left\{ \nu \in \mathbb {Z} ^{\operatorname {half}}\mid \nu \ge \mu \right\} $$

note that $d\in \left\{ 2,3,\ldots \right\}$ is equivalent to $\nu \in \mathbb {Z}^{\operatorname {half}}$.

Let the functions $r,r_{+}:\mathbb {R}^{d}\times \mathbb {R}^{d}\rightarrow \mathbb {R}$ be defined by

2.3 $$\begin{aligned} r\left( \textbf{x},\textbf{y}\right) :=\left\| \textbf{x}-\textbf{y} \right\| \quad {\text {and}}\quad r_{+}\left( \textbf{x},\textbf{y}\right) :=r\left( \textbf{x},\textbf{y}\right) +\beta \left( x_{d}-y_{d}\right) . \end{aligned}$$

If the second argument $\textbf{y}$ is zero, we use with a small abuse of notation the shorthand

2.4 $$\begin{aligned} r\left( \textbf{z}\right) :=r\left( \textbf{z},{\textbf {0}}\right) ,\quad r_{+}\left( \textbf{z}\right) :=r_{+}\left( \textbf{z},{\textbf {0}}\right) , \end{aligned}$$

and set for $\textbf{z}^{\prime }:=\left( z_{j}\right) _{j=1}^{d-1}$

2.5 $$\begin{aligned} y\left( \mathbf {z,\cdot }\right) :[ z_{d},\infty [ \rightarrow [ 0,\infty [ ,\qquad y\left( \textbf{z},t\right) :=-r_{+}\left( \textbf{z}\right) +\beta t+\mu \left( \textbf{z}^{\prime },t\right) \end{aligned}$$

with the function $\mu \left( \textbf{z}^{\prime },\cdot \right) :[ z_{d},\infty [ \rightarrow [ \left\| \textbf{z}\right\| ,\infty [$ given by

2.6 $$\begin{aligned} \mu \left( \textbf{z}^{\prime },t\right) :=\sqrt{\left\| \textbf{z} ^{\prime }\right\| ^{2}+t^{2}}. \end{aligned}$$

The derivative of *y* satisfies

2.7 $$\begin{aligned} \frac{\partial y\left( \textbf{z},t\right) }{\partial t}=\beta +\frac{t}{\mu \left( \textbf{z}^{\prime },t\right) }>0 \end{aligned}$$

so that $y\left( \textbf{z},\cdot \right)$ maps the interval $[ z_{d},\infty [$ strictly increasing onto [0,∞[. Its inverse

2.8 $$\begin{aligned} t\left( \textbf{z},\cdot \right) :[ 0,\infty [ \rightarrow [ z_{d},\infty [ \end{aligned}$$

is also strictly increasing. The derivative $\partial t\left( \textbf{z},y\right) /\partial y$ can be expressed by using (2.7):

2.9 $$\begin{aligned} \frac{\partial t\left( \textbf{z},y\right) }{\partial y}=\frac{\tilde{\mu }\left( \textbf{z},y\right) }{t\left( \textbf{z},y\right) +\beta \tilde{\mu }\left( \textbf{z},y\right) }, \end{aligned}$$

where

2.10 $$\begin{aligned} \tilde{\mu }\left( \textbf{z},y\right) :=\mu \left( \textbf{z}^{\prime },t\left( \textbf{z},y\right) \right) \quad {\text {and}}\quad \frac{\partial \tilde{\mu }\left( \textbf{z},y\right) }{\partial y}=\frac{t\left( \textbf{z},y\right) }{t\left( \textbf{z},y\right) +\beta \tilde{\mu }\left( \textbf{z},y\right) }>0. \end{aligned}$$

In the following, the shorthands

2.11 $$\begin{aligned} t=t\left( \textbf{z},y\right) ,\quad \tilde{\mu }=\tilde{\mu }\left( \textbf{z},y\right) \end{aligned}$$

will be used. A key role for the representation of the Green’s function will be played by the functions

2.12 $$\begin{aligned} \sigma _{\nu }\left( r,z\right) :=\frac{z-\beta r}{z+\beta r}g_{\nu }\left( r\right) \quad {\text {and}}\quad \psi _{\nu ,s}\left( \textbf{z}\right) :=\frac{1}{s}\int \limits _{0}^{\infty }\operatorname {e}\nolimits ^{-sy}q_{\nu }\left( \textbf{z},y\right) dy \end{aligned}$$

with

2.13 $$\begin{aligned} q_{\nu }\left( \textbf{z},y\right) :=\frac{d}{dy}\left( \frac{\operatorname {e}\nolimits ^{s\tilde{\mu }}K_{\nu +1/2}\left( s\tilde{\mu }\right) }{\left( t+\beta \tilde{\mu }\right) \left( s\tilde{\mu }\right) ^{\nu -1/2}}\right) . \end{aligned}$$

The following representation of the half-space Green’s function follows from [23, Thm. 3.1 and Rem. 3.2]:

### Definition 2.2

Let $d\in \left\{ 2,\ldots \right\}$ denote the spatial dimension. The Green’s function for the acoustic half-space problem with impedance boundary conditions, i.e. the solution of (2.1), is given by:

2.14 $$\begin{aligned} G_{\operatorname {half}}\left( \textbf{x},\textbf{y}\right) :=G_{\operatorname {illu}}\left( \textbf{x}-\textbf{y}\right) +G_{\operatorname {refl}}\left( \textbf{x}-\textbf{Ry}\right) +G_{\operatorname {imp}}\left( \textbf{x}-\textbf{Ry}\right) , \end{aligned}$$

where, with $$\nu =\left( d-3\right) /2$$,

$$ G_{\operatorname {illu}}\left( \textbf{z}\right) :=g_{\nu }\left( \left\| \textbf{z}\right\| \right) ,\quad G_{\operatorname {refl}}\left( \textbf{z}\right) :=\sigma _{\nu }\left( \left\| \textbf{z}\right\| ,z_{d}\right) ,\quad G_{\operatorname {imp}}\left( \textbf{z}\right) :=-\frac{\beta }{\pi }\left( \frac{s^{2}}{2\pi }\right) ^{\nu +1/2} \operatorname {e}\nolimits ^{-s\left\| \textbf{z}\right\| }\psi _{\nu ,s}\left( \textbf{z}\right) . $$

In [23], it is proved that $G_{\operatorname {half}}$ satisfies problem (2.1), and thus, the name Green’s function is justified.

## Slowly varying functions and their approximation

In this section, we will first prove that the half-space Green’s function in (2.14) can be split into a sum of (three) terms each of which can be written as a product of the form $\operatorname {e}^{-s\tau _{\ell }\left( \textbf{x},\textbf{y}\right) }g_{\ell }\left( \textbf{x},\textbf{y}\right)$, where $\tau _{\ell }$ denotes an *eikonal* and the function $g_{\ell }$ is *slowly varying.* To make the meaning of these notions precise, some preparations are necessary. For two points $\textbf{x},\textbf{y}$ in the domain, the eikonal $\tau _{\ell }\left( \textbf{x},\textbf{y}\right)$ is the length of a possible path of light (in the limit of geometric optics) for a ray emitted at $\textbf{y}$ and received at $\textbf{x}$. In this way, the eikonal for the direct ray between $\textbf{x}$ and $\textbf{y}$ is given by $\tau _{\operatorname {illu}}\left( \textbf{x},\textbf{y}\right) :=\left\| \textbf{x}-\textbf{y}\right\|$ and for the reflected ray by $\tau _{\operatorname {refl}}\left( \textbf{x},\textbf{y}\right) :=\left\| \textbf{x}-\textbf{Ry}\right\|$. For the half-space Green’s function, we set for $\textbf{x},\textbf{y}\in H_{+}$,

3.1 $$\begin{aligned} \textbf{z}:=\textbf{x}-\textbf{y},\quad r:=r\left( \textbf{x},\textbf{y} \right) =\left\| \textbf{z}\right\| ,\quad \textbf{z}_{-}:=\textbf{x} -\textbf{Ry}{,\quad }r_{-}:=r\left( \textbf{x},\textbf{Ry}\right) =\left\| \textbf{z}_{-}\right\| \end{aligned}$$

and define

3.2a $$\begin{aligned} \Theta _{\nu ,s}^{\operatorname {illu}}\left( \textbf{x},\textbf{y}\right)&:=\operatorname {e}\nolimits ^{sr}g_{\nu }\left( r\right) , \end{aligned}$$

3.2b $$\begin{aligned} \Theta _{\nu ,s}^{\operatorname {refl}}\left( \textbf{x},\textbf{y}\right)&:=\operatorname {e}\nolimits ^{sr_{-}}\sigma _{\nu }\left( r_{-},x_{d} +y_{d}\right) , \end{aligned}$$

3.2c $$\begin{aligned} \Theta _{\nu ,s}^{\operatorname {imp}}\left( \textbf{x},\textbf{y}\right)&:=-\frac{\beta }{\pi }\left( \frac{s^{2}}{2\pi }\right) ^{\nu +1/2}\psi _{\nu ,s}\left( \textbf{z}_{-}\right) . \end{aligned}$$

With this notation at hand, the half-space Green’s function can be written in the form

3.3 $$\begin{aligned} G_{\operatorname {half}}:=\operatorname {e}\nolimits ^{-s\tau _{\operatorname {illu}}}\Theta _{\nu ,s}^{\operatorname {illu}} +\operatorname {e}\nolimits ^{-s\tau _{\operatorname {refl}}}\left( \Theta _{\nu ,s}^{\operatorname {refl}}+\Theta _{\nu ,s}^{\operatorname {imp}}\right) . \end{aligned}$$

The functions in (3.2) are collected in the families^1^

3.4a $$\begin{aligned} \mathcal {F}_{\nu }^{\operatorname {illu}}&:=\left\{ \Theta _{\nu ,s}^{\operatorname {illu}}:\mathbb {R}^{d}\times \mathbb {R}^{d}\rightarrow \mathbb {C}\mid s\in \overset{\bullet }{\mathbb {C}}_{\ge 0}\right\} , \end{aligned}$$

3.4b $$\begin{aligned} \mathcal {F}_{\nu }^{\operatorname {refl}}&:=\left\{ \Theta _{\nu ,s}^{\operatorname {refl}}:H_{+}\times H_{+}\rightarrow \mathbb {C}\mid s\in \overset{\bullet }{\mathbb {C}}_{\ge 0}\right\} , \end{aligned}$$

3.4c $$\begin{aligned} \mathcal {F}_{\nu }^{\operatorname {imp}}&:=\left\{ \Theta _{\nu ,s}^{\operatorname {imp}}:H_{+}\times H_{+}\rightarrow \mathbb {C}\mid s\in \overset{\bullet }{\mathbb {C}}_{\ge 0}\right\} , \end{aligned}$$

and our goal is to prove that these families are slowly varying, a notion which will be introduced next.

We define families of slowly varying functions via polynomial approximability, which is the key mechanism of most numerical approximation methods. Loosely speaking a family of functions depending on a (frequency) parameter, say *s*, is slowly varying if polynomial approximation converges exponentially (with no or weak dependence on *s*). Polynomial approximability on a real interval for *analytic functions* relies on the modulus of these functions on *Bernstein ellipses* encircling the interval. We start this section with some elementary geometric considerations and recall the tensor Chebyshev interpolation.

For $\textbf{a}=\left( a_{i}\right) _{i=1}^{d}\in \mathbb {R}^{d}$ and $\textbf{b}=\left( b_{i}\right) _{i=1}^{d}\in \mathbb {R}^{d}$ with $-\infty<a_{i}<b_{i}<\infty$ for all $i\in \left\{ 1,2,\ldots ,d\right\}$, the corresponding cuboid is

$$ \left[ \textbf{a},\textbf{b}\right] := \mathop {\times }\limits _{j=1}^{d}\left[ a_{j},b_{j}\right] . $$

We recall the tensor Chebyshev interpolation on a block of cuboids $\left[ \textbf{a},\textbf{b}\right] \times \left[ \textbf{c},\textbf{d}\right]$. Let $\hat{\xi }_{i,m}$, $0\le i\le m,$ denote the Chebyshev points in the unit interval $\left[ -1,1\right]$, and let $\hat{L}_{i,m}$ be the corresponding Lagrange polynomials. The tensor version employs the index set

$$ \iota _{m}:=\left\{ 0,1,\ldots ,m\right\} ^{d} $$

and is given, for ${\boldsymbol{\mu }} \in \iota _{m}$, by $\widehat{ {\boldsymbol{\xi }} }_{ {\boldsymbol{\mu }},m}:=\left( \hat{\xi }_{\mu _{1},m},\hat{\xi }_{\mu _{2},m},\ldots ,\hat{\xi } _{\mu _{d},m}\right) ^{\intercal }$ and^2^$\hat{L}_{ {\boldsymbol{\mu }},m}= {\displaystyle \bigotimes \limits _{\ell =1}^{d}} \hat{L}_{\mu _{\ell },m}$. For a box $\left[ \textbf{a},\textbf{b}\right]$, let $\chi _{\left[ \textbf{a},\textbf{b}\right] }:[-1,1]^{d}\rightarrow [\textbf{a},\textbf{b}]$ denote an affine pullback to the reference cuboid $\left[ -1,1\right] ^{d}$. Then, the tensorized Chebyshev nodal points of order *m* scaled to the box $\left[ \textbf{a},\textbf{b}\right]$ are given by ${\boldsymbol{\xi }} _{ \boldsymbol{\mu },m}^{\left[ \textbf{a},\textbf{b}\right] }:=\chi _{\left[ \textbf{a},\textbf{b}\right] }\left( \widehat{ \boldsymbol{\xi } }_{ \boldsymbol{\mu },m}\right)$ and $L_{ \boldsymbol{\mu },m}^{\left[ \textbf{a},\textbf{b}\right] }:=\hat{L}_{ \boldsymbol{\mu },m}\circ \chi _{\left[ \textbf{a},\textbf{b}\right] }^{-1}$, for all $\boldsymbol{\mu } \in \mathbb {\iota }_{m}$.

### Definition 3.1

Let $\left[ \textbf{a},\textbf{b}\right] ,\left[ \textbf{c},\textbf{d}\right]$ be two axes-parallel cuboids. The *tensor Chebyshev interpolation operator* $\Pi _{m}^{\left[ \textbf{a},\textbf{b} \right] \times \left[ \textbf{c},\textbf{d}\right] }:C^{0}\left( \left[ \textbf{a},\textbf{b}\right] \times \left[ \textbf{c},\textbf{d}\right] \right) \rightarrow {\mathcal {Q}}_{m}:=\operatorname {span}\{L_{ \boldsymbol{\mu },m}^{\left[ \textbf{a},\textbf{b}\right] }\otimes L_{ \boldsymbol{\nu },m}^{\left[ \textbf{c},\textbf{d}\right] }\,|\, \boldsymbol{\mu },\boldsymbol{\nu } \in \{0,\ldots ,m\}^{d}\}$ of degree $m\in \mathbb {N}$ is given by

$$ \Pi _{m}^{\left[ \textbf{a},\textbf{b}\right] \times \left[ \textbf{c},\textbf{d}\right] }k:=\sum _{ \boldsymbol{\mu },\boldsymbol{\nu } \in \iota _{m}}k\left( \boldsymbol{\xi } _{ \boldsymbol{\mu },m}^{\left[ \textbf{a},\textbf{b}\right] },\boldsymbol{\xi } _{ \boldsymbol{\nu },m}^{\left[ \textbf{c},\textbf{d}\right] }\right) L_{ \boldsymbol{\mu },m}^{\left[ \textbf{a},\textbf{b}\right] }\otimes L_{ \boldsymbol{\nu },m}^{\left[ \textbf{c},\textbf{d}\right] }. $$

For the analysis of the approximation error, we will employ classical error estimates for Chebyshev interpolation of analytic functions (see [13]). This is done by estimating the modulus of analytic functions on *Bernstein ellipses*, and we recall their basic properties.

Let $-\infty<a<b<\infty$ and consider the real interval $\left[ a,b\right]$. Let $\mathcal {E}_{a,b}^{\rho }\subset \mathbb {C}$ be the closed ellipse with focal points *a*, *b* and semimajor/semiminor axes $\overline{a}$, $\overline{b}$ given by

3.5 $$\begin{aligned} \bar{a}=\frac{\rho ^{2}+\left( \frac{b-a}{2}\right) ^{2}}{2\rho }\ge \frac{b-a}{2},\qquad \bar{b}=\frac{\rho ^{2}-\left( \frac{b-a}{2}\right) ^{2} }{2\rho }\ge 0, \end{aligned}$$

where the estimates become an equality if and only the ellipse collapses to the interval: $\left[ a,b\right] =\mathcal {E}_{a,b}^{\rho }$ for $\rho =\left( b-a\right) /2$. The sum of the half-axes is given by $\rho =\bar{a}+\bar{b}$.

For $j\in \left\{ 1,2,\ldots ,d\right\}$, the ellipses $\mathcal {E} _{a_{j},b_{j}}^{\rho _{j}}$ refer to the coordinate intervals $I_{j}:=\left[ a_{j},b_{j}\right]$ and the semi-axes sums form the vector $\boldsymbol{\rho }:=\left( \rho _{i}\right) _{i=1}^{d}$. For $j\in \left\{ 1,2,\ldots ,d\right\}$, we set

3.6 $$\begin{aligned} \overrightarrow{\mathcal {E}}_{\textbf{a},\textbf{b}}^{j}\left( \boldsymbol{\rho } \right) :=I_{1}\times I_{2}\times \cdots \times I_{j-1}\times \mathcal {E} _{a_{j},b_{j}}^{\rho _{j}}\times I_{j+1}\times \cdots \times I_{d} \end{aligned}$$

and define

3.7 $$\begin{aligned} \overrightarrow{\mathcal {E}}_{\textbf{a},\textbf{b},\textbf{c},\textbf{d} }\left( \boldsymbol{\rho } _{1},\boldsymbol{\rho } _{2}\right) := {\displaystyle \bigcup \limits _{j=1}^{d}} \left( \left( \overrightarrow{\mathcal {E}}_{\textbf{a},\textbf{b}} ^{j}\left( \boldsymbol{\rho } _{1}\right) \times \left[ \textbf{c},\textbf{d}\right] \right) \cup \left( \left[ \textbf{a},\textbf{b}\right] \times \overrightarrow{\mathcal {E} }_{\textbf{c},\textbf{d}}^{j}\left( \boldsymbol{\rho } _{2}\right) \right) \right) . \end{aligned}$$

For univariate functions that are analytic in (closed) Bernstein ellipses about some real interval, it can be proved (see [13]) that Chebyshev interpolation converges exponentially. Our componentwise extension to $\overrightarrow{\mathcal {E}}_{\textbf{a},\textbf{b},\textbf{c},\textbf{d} }\left( \boldsymbol{\rho } _{1},\boldsymbol{\rho } _{2}\right)$ is chosen such that a tensor argument yields exponential convergence for Chebyshev interpolation on axes-parallel cuboids. The proof of the following proposition can be found in [33, Thm. 7.3.6].

### Proposition 3.2

([33, Thm. 7.3.6])Let $\left[ \textbf{a},\textbf{b}\right]$, $\left[ \textbf{c},\textbf{d} \right]$ be axes-parallel cuboids. Assume that the function $k\in C^{0}\left( \left[ \textbf{a},\textbf{b}\right] \times \left[ \textbf{c},\textbf{d}\right] \right)$ can be extended analytically^3^ to $\overrightarrow{\mathcal {E}}_{\textbf{a},\textbf{b},\textbf{c},\textbf{d} }\left( \boldsymbol{\rho } _{1},\boldsymbol{\rho } _{2}\right)$ with $\left( \boldsymbol{\rho } _{1}\right) _{i}>\left( b_{i}-a_{i}\right) /2$ and $\left( \boldsymbol{\rho } _{2}\right) _{i}>\left( d_{i}-c_{i}\right) /2$, $1\le i\le d$ (and is denoted again by *k*). Then, the Chebyshev interpolant in Definition 3.1 satisfies the error estimate

$$ \left\| k-\Pi _{m}^{\left[ \textbf{a},\textbf{b}\right] \times \left[ \textbf{c},\textbf{d}\right] }k\right\| _{C^{0}\left( \left[ \textbf{a},\textbf{b}\right] \times \left[ \textbf{c},\textbf{d}\right] \right) }\le C_{\gamma }\gamma ^{-m}M_{ \boldsymbol{\rho } _{1},\boldsymbol{\rho } _{2}}\left( k\right) $$

with the relative extension parameter

$$ \gamma :=\min \left\{ \min \left\{ \frac{2\left( \boldsymbol{\rho } _{1}\right) _{j}}{b_{j}-a_{j}}:1\le j\le d\right\} ,\min \left\{ \frac{2\left( \boldsymbol{\rho } _{2}\right) _{j}}{d_{j}-c_{j}}:1\le j\le d\right\} \right\} $$

and

$$ M_{ \boldsymbol{\rho } _{1},\boldsymbol{\rho } _{2}}\left( k\right) :=\max _{\left( \textbf{x},\textbf{y}\right) \in \overrightarrow{\mathcal {E}}_{\textbf{a},\textbf{b},\textbf{c},\textbf{d} }\left( \boldsymbol{\rho } _{1},\boldsymbol{\rho } _{2}\right) }\left| k\left( \textbf{x},\textbf{y}\right) \right| . $$

The constant $C_{\gamma }$ is given by:

$$ C_{\gamma }:=\sqrt{d}2^{d+3/2}\left( 1-\gamma ^{-2}\right) ^{-d}. $$

In our application, we consider functions that depend on a frequency parameter *s*. We define the notion “κ-slowly varying” for such families of functions and first introduce an *admissibility condition*.

### Definition 3.3

Let $0<\eta _{0}$ be fixed. For $\eta \in ] 0,\eta _{0}]$, two subsets *B*,$C\subset \mathbb {R}^{d}$ are η-*admissible* if

3.8 $$\begin{aligned} \max \left\{ \operatorname {diam}B,\operatorname {diam}C\right\} \le \eta \operatorname {dist}\left( B,C\right) . \end{aligned}$$

In the first expression in (3.3), the first prefactor is oscillatory if $\operatorname {Im}s\ne 0$ and the product “$\left( \operatorname {Im}s\right) \times \left\| \textbf{x}-\textbf{y} \right\|$” is large. More specifically, we say that the triplet“$(s,\textbf{x},\textbf{y})$” belongs to the *slowly oscillatory regime* if $\left| s\right| \left\| \textbf{x} -\textbf{y}\right\| \le 1$ while it belongs to the oscillatory regime if $|s| \Vert {\textbf{x}-\textbf{y}}\Vert > 1$. These different ranges are reflected in Definition 3.4 of *algebraically bounded functions* being employed in the definition of a κ-slowly varying family of functions.

### Definition 3.4

*(algebraically bounded)*Let $\omega \subset \mathbb {C}$. A function $g:\omega \rightarrow \mathbb {R}_{\ge 0}$ is *algebraically bounded* for growth parameters $\boldsymbol{\alpha } =\left( \alpha _{1},\alpha _{2}\right) \in \mathbb {R}^{2}$ and some C≥0 if

$$ g\left( \zeta \right) \le C\times {\left\{ \begin{array}{ll} \left| \zeta \right| ^{-\alpha _{1}} \, {\text {if }}\left| \zeta \right| \ge 1,\\ \left| \zeta \right| ^{-\alpha _{2}} \, {\text {if }}\left| \zeta \right| \le 1, \end{array}\right. } \quad \forall \zeta \in \omega . $$

The definition of a κ-slowly varying family of functions relies on the holomorphic extensibility of functions from coordinate intervals to complex ellipses. While the parameter η (cf. (3.8)) is related to the admissibility condition for pairs of cuboids, a further parameter κ>0 measures the relative size of the extended region: We define for $j\in \left\{ 1,2,\ldots ,d\right\} :$

3.9 $$\begin{aligned} \left( \boldsymbol{\rho } _{1}\right) _{j}:=\left( \boldsymbol{\rho } _{1}\right) _{j}\left( \kappa \right) :=\frac{b_{j}-a_{j}}{2}\left( 1+\frac{2\kappa }{\eta }\right) ,{\quad }\left( \boldsymbol{\rho } _{2}\right) _{j}:=\left( \boldsymbol{\rho } _{2}\right) _{j}\left( \kappa \right) :=\frac{d_{j}-c_{j}}{2}\left( 1+\frac{2\kappa }{\eta }\right) \end{aligned}$$

and use the shorthands

$$ \mathcal {E}_{a_{j},b_{j}}\left( \kappa \right) :=\mathcal {E}_{a_{j},b_{j} }^{({\boldsymbol{\rho }}_{1})_{j}\left( \kappa \right) },\quad \overrightarrow{\mathcal {E}}_{\textbf{a},\textbf{b}}^{j}\left( \kappa \right) :=\overrightarrow{\mathcal {E}}_{\textbf{a},\textbf{b}}^{j}\left( \boldsymbol{\rho } _{1}\left( \kappa \right) \right) ,\quad \overrightarrow{\mathcal {E} }_{\textbf{a},\textbf{b},\textbf{c},\textbf{d}}\left( \kappa \right) :=\overrightarrow{\mathcal {E}}_{\textbf{a},\textbf{b},\textbf{c},\textbf{d} }\left( \boldsymbol{\rho } _{1}\left( \kappa \right) ,\boldsymbol{\rho } _{2}\left( \kappa \right) \right) . $$

### Definition 3.5

*(slowly varying family of functions)*Let $0<\eta \le \eta _{0}$, κ>0, and $\omega \subset \mathbb {C}$ be given. For fixed $D\subset \mathbb {R}^{d}$, consider a family of functions

$$ \mathcal {F}:=\left\{ F_{s}:D\times D\rightarrow \mathbb {C}\mid s\in \omega \right\} $$

along with an algebraically bounded *reference function* $\lambda :\mathbb {C}\rightarrow \mathbb {R}_{>0}$ and constants $C_{\mathcal {F}}>0$, $\tau \in \mathbb {R}$.

The family $\mathcal {F}$ is κ-*slowly varying* (with respect to λ, $C_{\mathcal {F}}$, and τ) if for any block of η-admissible cuboids $B:=\left[ \textbf{a},\textbf{b}\right] \times \left[ \textbf{c},\textbf{d}\right] \subset D\times D$ with distance $\delta :=\operatorname {dist}\left( \left[ \textbf{a},\textbf{b}\right] ,\left[ \textbf{c},\textbf{d}\right] \right) >0$ and any $F_{s}\in {\mathcal {F}}$, the function $\left. F_{s}\right| _{B}$ can be extended analytically to $\overrightarrow{\mathcal {E}}_{\textbf{a},\textbf{b},\textbf{c},\textbf{d} }\left( \kappa \right)$ and satisfies

$$ \max _{\left( \textbf{x},\textbf{y}\right) \in \overrightarrow{\mathcal {E} }_{\textbf{a},\textbf{b},\textbf{c},\textbf{d}}\left( \kappa \right) }\left| F_{s}\left( \textbf{x},\textbf{y}\right) \right| \le C_{\mathcal {F}}\left| s\right| ^{\tau }\lambda \left( \left| s\right| \delta \right) . $$

For κ-slowly varying function families, the tensor Chebyshev interpolation converges exponentially as can be seen from the following corollary, which follows from Proposition 3.2.

### Corollary 3.6

Let η>0, κ>0, and $\omega \subset \mathbb {C}$ be given. For fixed $D\subseteq \mathbb {R}^{d}$, consider a family of functions

$$ \mathcal {F}:=\left\{ k_{s}:D\times D\rightarrow \mathbb {C}\mid s\in \omega \right\} $$

that is κ-*slowly varying* with reference function λ and constants $C_{\mathcal {F}}$, τ as in Definition 3.5.

Then, for any $s\in \omega$ and any block of η-admissible cuboids $\left[ \textbf{a},\textbf{b}\right] \times \left[ \textbf{c},\textbf{d}\right] \subset D\times D$ it holds:

$$ \left\| k_{s}-\Pi _{m}^{\left[ \textbf{a},\textbf{b}\right] \times \left[ \textbf{c},\textbf{d}\right] }k_{s}\right\| _{C^{0}\left( \left[ \textbf{a},\textbf{b}\right] \times \left[ \textbf{c},\textbf{d}\right] \right) }\le C_{\mathcal {F}}\left| s\right| ^{\tau }\lambda \left( \left| s\right| \delta \right) C_{\gamma }\gamma ^{-m}\quad \forall m\in \mathbb {N}_{0} $$

with $$\gamma =1+2\kappa /\eta $$ and $$\delta :=\operatorname {dist}\left( \left[ \textbf{a},\textbf{b}\right] ,\left[ \textbf{c},\textbf{d}\right] \right) $$.

Since the function λ is algebraically bounded, an accuracy $\left\| k_{s}-\Pi _{m}^{\left[ \textbf{a},\textbf{b}\right] \times \left[ \textbf{c},\textbf{d}\right] }k_{s}\right\| _{C^{0}\left( \left[ \textbf{a},\textbf{b}\right] \times \left[ \textbf{c},\textbf{d}\right] \right) }\le \varepsilon$ for given ε>0 is reached for a polynomial degree *m* which depends only linearly on $\left| \ln \varepsilon \right|$, $\left| \ln \left| s\right| \right|$, τ, and $\left| \ln \left| s\delta \right| \right|$.

### Lemma 3.7

Consider a family of functions $\mathcal {F}:=\left\{ F_{s}:D\times D\rightarrow \mathbb {C}\mid s\in \omega \right\}$ that is κ-*slowly varying* with reference function λ as in Definition 3.5 Then for every η-admissible cuboid ${\textbf{B}}=[\textbf{a},\textbf{b}]\times [\textbf{c},\textbf{d}]\subset D\times D$ there is a complex neighbourhood ${\mathcal {B}}\subset {\mathbb {C}}^{d}\times {\mathbb {C}}^{d}$ of ${\textbf{B}}$ such that every $F_{s}\in {\mathcal {F}}$ can be extended analytically to ${\mathcal {B}}$. Furthermore, given T>0 there are constants $C_{1}$, $C_{2}$ depending solely on *T* such that for $\kappa /\eta \le T$ there holds upon setting $\Theta :=\min _{j=1,\ldots ,d}\min \{(b_{j}-a_{j}),(d_{j}-c_{j})\}$

3.10 $$\begin{aligned} \Vert \partial _{x}^{\boldsymbol{\mu }}\partial _{y}^{\boldsymbol{\nu }}F_{s} \Vert _{C^{0}(\textbf{B})}\le C_{1}C_{\mathcal {F}}|s|^{\tau }\lambda (\left| s\right| \delta ){\boldsymbol{\mu }}!{\boldsymbol{\nu }}!\left( C_{2}\left( \eta /\kappa \right) ^{2}\Theta \right) ^{|{\boldsymbol{\mu }} |+|{\boldsymbol{\nu }}|}. \end{aligned}$$

### Proof

By assumption, the function $$F_{s}$$ can be extended analytically in each variable. For quantitative bounds, we have to consider $$\operatorname {dist}([a_{j},b_{j}],\partial \mathcal {E}_{a_{j},b_{j}}(\kappa ))$$. For small $$\kappa /\eta $$, one has $$\operatorname {dist}([a_{j},b_{j}],\partial \mathcal {E}_{a_{j},b_{j}}(\kappa ))\gtrsim (b_{j}-a_{j})\left( \frac{\kappa }{\eta }\right) ^{2}$$ with implied constant independent of $$a_{j} $$, $$b_{j}$$, $$\kappa /\eta $$. An analogous result holds for the intervals $$[c_{j},d_{j}]$$. Hence, Cauchy’s integral theorem applied to any $$x_{j} \in \left[ a_{j},b_{j}\right] \in \mathcal {E}_{a_{j},b_{j}}(\kappa )$$ (any $$y_{j}\in \left[ c_{j},d_{j}\right] \subset \mathcal {E}_{c_{j},d_{j}}(\kappa )$$) allows us to control the partial derivatives $$\partial _{x_{j}}^{\mu _{j}}$$, $$\partial _{y_{j}}^{\nu _{j}}$$ of $$F_{s}$$ in the stated fashion. The set $$\mathcal {B}$$ may be taken as $$ {\displaystyle \bigcup _{\textbf{x}\in \textbf{B}}} B_{r}\left( \textbf{x}\right) \subset \mathbb {C}^{d}\times \mathbb {C}^{d}$$ for $$r=1/\left( C_{2}\left( \eta /\kappa \right) ^{2}\Theta \right) .$$ □

## Analysis of the families $\Theta _{\nu ,s}^{\operatorname {illu} },\Theta _{\nu ,s}^{\operatorname {refl}},\Theta _{\nu ,s}^{\operatorname {imp}}$

In this chapter, we will prove that the families of functions $\Theta _{\nu ,s}^{\operatorname {illu}}$, $\Theta _{\nu ,s}^{\operatorname {refl}}$, $\Theta _{\nu ,s}^{\operatorname {imp}}$ in (3.2) are κ-slowly varying. First, we present a majorant for the modified Bessel function $K_{\nu }\left( z\right)$ which is uniform for all $z\in \mathbb {C}\backslash \mathbb {R}_{\le 0}$ (see §4.1). Since the functions $\Theta _{\nu ,s}^{\operatorname {illu}}$ and $\Theta _{\nu ,s}^{\operatorname {refl}}$ depend on the Euclidean norms $\left\| \textbf{x}-\textbf{y}\right\|$ and $\left\| \textbf{x}-\textbf{Ry} \right\|$, we will derive estimates for the holomorphic norm extension to complex ellipses (see §4.2). These results are then combined to prove that $\Theta _{\nu ,s}^{\operatorname {illu}}$ and $\Theta _{\nu ,s}^{\operatorname {refl}}$ are κ-slowly varying (§4.3). Finally, in Sect. 4.4 we show that $\Theta _{\nu ,s}^{\operatorname {imp}}$ is κ-slowly varying and the main theorem (Thm. 4.2) of this paper follows.

### Notation 4.1

$C_{\nu }$ is a constant depending only on $\nu \in \mathbb {Z} ^{\operatorname {half}}$ and may change its value in each appearance.

$C_{\nu ,\beta }$ depends on $\nu \in \mathbb {Z}^{\operatorname {half}}$ and is a continuous function of the impedance parameter β>0. It may exhibit algebraic or logarithmic singularities at the endpoints $\beta \in \left\{ 0,\infty \right\}$ whose strength depends on ν. It may change its value in each appearance.

For the formulation of the main theorem, two (similar) functions $M_{\mu }:\mathbb {R}_{>0}\rightarrow \mathbb {R}_{>0}$ and $W_{\mu }:\mathbb {R} _{>0}\rightarrow \mathbb {R}_{>0}$ are needed:

4.1a $$\begin{aligned} M_{\mu }\left( r\right)&:=\left\{ \begin{array}{ll} r^{-1/2} &  {\text {for }}r\ge 1,\\ r^{-\mu } &  {\text {for }}0<r\le 1\wedge \mu \in \mathbb {Z}_{\ge \frac{1}{2} }^{\operatorname {half}},\\ 1+\left| \ln r\right| &  {\text {for }}0<r\le 1\wedge \mu =0, \end{array} \right. \end{aligned}$$

4.1b $$\begin{aligned} W_{\mu }\left( r\right)&:=\left\{ \begin{array}{ll} r^{-1/2} &  {\text {for }}r\ge 1,\\ r^{-\mu } &  {\text {for }}0<r\le 1\wedge \mu \in \mathbb {Z}_{\ge \frac{1}{2} }^{\operatorname {half}},\\ 1+\ln ^{2}r &  {\text {for }}0<r\le 1\wedge \mu =0. \end{array} \right. \end{aligned}$$

### Theorem 4.2

Let $0<\eta \le \eta _{0}$ be as in Definition 3.3, $\nu \in \mathbb {Z}^{\operatorname {half}},$ and let β>0 denote the impedance parameter in (2.1). The families of functions $\mathcal {F} _{\nu }^{\operatorname {illu}}$, $\mathcal {F}_{\nu }^{\operatorname {refl}}$, $\mathcal {F}_{\nu }^{\operatorname {imp}}$ in (3.4) are κ-slowly varying:

1. For any $\kappa \in ] 0,\min \left\{ \frac{1}{6},\frac{1}{3\beta },\frac{\beta }{6}\right\} [$, any η-admissible block $B=\left[ \textbf{a},\textbf{b}\right] \times \left[ \textbf{c},\textbf{d}\right] \subset H_{+}\times H_{+}$ and $\delta :=\operatorname {dist}\left( \left[ \textbf{a},\textbf{b}\right] ,\left[ \textbf{c},\textbf{d}\right] \right)$ it holds
  4.2a $$\begin{aligned} \max _{\left( \textbf{x},\textbf{y}\right) \in \overrightarrow{\mathcal {E} }_{\textbf{a},\textbf{b},\textbf{c},\textbf{d}}\left( \kappa \right) }\left| \Theta _{\nu ,s}^{\operatorname {illu}}\left( \textbf{x} ,\textbf{y}\right) \right|&\le C_{\nu }\left( \frac{\left| s\right| }{\delta }\right) ^{\nu +1/2}M_{\nu +1/2}\left( \left| s\right| \delta \right) , \end{aligned}$$
  4.2b $$\begin{aligned} \max _{\left( \textbf{x},\textbf{y}\right) \in \overrightarrow{\mathcal {E} }_{\textbf{a},\textbf{b},\textbf{c},\textbf{d}}\left( \kappa \right) }\left| \Theta _{\nu ,s}^{\operatorname {refl}}\left( \textbf{x} ,\textbf{y}\right) \right|&\le C_{\nu ,\beta }\left( \frac{\left| s\right| }{\delta }\right) ^{\nu +1/2}M_{\nu +1/2}\left( \left| s\right| \delta \right) \end{aligned}$$
  with $M_{\nu +1/2}$ as in (4.1a). The constants $C_{\mathcal {F}}$ and τ in Definition 3.5 for the first case (4.2a) can be chosen as $C_{\mathcal {F}}=C_{\nu }$ and $\tau =2\nu +1$ and in the second one (4.2b) as $C_{\mathcal {F}}:=C_{\nu ,\beta }$ and $\tau =2\nu +1$. The reference function λ can be chosen for both cases as
  4.3 $$\begin{aligned} \lambda :\overset{\bullet }{\mathbb {C}}_{\ge 0}\rightarrow \mathbb {R}_{>0} \quad \lambda \left( \zeta \right) :=\left\{ \begin{array}{ll} \left| \zeta \right| ^{-\nu -1} &  {\text {for }}\left| \zeta \right| \ge 1,\\ \left| \zeta \right| ^{-2\nu -1} &  {\text {for }}0<\left| \zeta \right| \le 1\wedge \nu \in \mathbb {Z}_{\ge 0}^{\operatorname {half}},\\ 1+\left| \ln \left| \zeta \right| \right| &  {\text {for }}0<\left| \zeta \right| \le 1\wedge \nu =-1/2. \end{array} \right. \end{aligned}$$
2. There exists a positive number $C_{\mathcal {E}}$ independent of all parameters and functions such that for any $\kappa \in ] 0,\frac{\beta ^{2}}{C_{\mathcal {E}}\left( 1+\beta \right) ^{3}}[$, any η-admissible block $B=\left[ \textbf{a},\textbf{b}\right] \times \left[ \textbf{c},\textbf{d}\right] \subset H_{+}\times H_{+}$ with $\delta :=\operatorname {dist}\left( \left[ \textbf{a},\textbf{b}\right] ,\left[ \textbf{c},\textbf{d}\right] \right)$, it holds
  $$ \max _{\left( \textbf{x},\textbf{y}\right) \in \overrightarrow{\mathcal {E} }_{\textbf{a},\textbf{b},\textbf{c},\textbf{d}}\left( \kappa \right) }\left| \Theta _{\nu ,s}^{\operatorname {imp}}\left( \textbf{x},\textbf{y}\right) \right| \le C_{\nu ,\beta }\left( \frac{\left| s\right| }{\delta }\right) ^{\nu +1/2}W_{\nu +1/2}\left( \left| s\right| \delta \right) $$
  with $W_{\mu }$ as in (4.1b). The constants $C_{\mathcal {F}}$ and τ in Definition 3.5 can be chosen as $C_{\mathcal {F} }:=C_{\nu ,\beta }$ and $\tau =2\nu +1$ and the reference function as
  $$ \lambda :\overset{\bullet }{\mathbb {C}}_{\ge 0}\rightarrow \mathbb {R}_{>0} \quad \lambda \left( \zeta \right) :=\left\{ \begin{array}{ll} \left| \zeta \right| ^{-\nu -1} &  {\text {for }}\left| \zeta \right| \ge 1,\\ \left| \zeta \right| ^{-2\nu -1} &  {\text {for }}0<\left| \zeta \right| \le 1\wedge \nu \in \mathbb {Z}_{\ge 0}^{\operatorname {half}},\\ 1+\ln ^{2}\left| \zeta \right| &  {\text {for }}0<\left| \zeta \right| \le 1\wedge \nu =-1/2. \end{array} \right. $$

The proof of this theorem is a consequence of Lemmata 4.8 and 4.12.

### A majorant for the Macdonald function $K_{\mu }$

In this section, we derive a majorant for the modified Bessel function of the second kind. For R>0, let $B_{R}:=\left\{ \zeta \in \mathbb {C}\mid \left| \zeta \right| \le R\right\}$.

#### Lemma 4.3

Let $\mu \in \mathbb {Z}_{\ge 0}^{\operatorname {half}}$. The modified Bessel function $K_{\mu }$ satisfies the estimates

1. 4.4a $$\begin{aligned} \left| \operatorname {e}\nolimits ^{z}K_{\mu }\left( z\right) \right| \le \sqrt{\frac{\pi }{2\left| z\right| }}\exp \left( \frac{\left| \mu ^{2}-\tfrac{1}{4}\right| }{\left| z\right| }\right) \quad \forall z\in \mathbb {C}\backslash \mathbb {R}_{\le 0}, \end{aligned}$$
2. 4.4b $$\begin{aligned} \left| \operatorname {e}\nolimits ^{z}\left( K_{\mu }\left( z\right) -K_{\mu +1}\left( z\right) \right) \right| \le \frac{C_{\mu }}{\left| z\right| ^{3/2}}\exp \left( \frac{C_{\mu }}{\left| z\right| }\right) \quad \forall z\in \mathbb {C}\backslash \mathbb {R}_{\le 0} \end{aligned}$$
  with $$C_{\mu }:=\sqrt{2\pi ^{3}}\left( \mu +1\right) ^{2}$$.
3. For any R>0, there exists a constant $C_{\mu }\left( R\right)$ depending only on μ and *R* such that the Bessel function $K_{\mu }$ satisfies:
  4.4c $$\begin{aligned} \left| \operatorname {e}\nolimits ^{z}K_{\mu }\left( z\right) \right| \le C_{\mu }\left( R\right) \times \left\{ \begin{array}{ll} \left( 1+\left| \ln \left| z\right| \right| \right) , &  \mu =0,\\ \left( \dfrac{\left| z\right| }{2}\right) ^{-\mu }, &  {\text {otherwise,}} \end{array} \right. \quad \forall z\in B_{R}\backslash \left( \left[ -R,0\right] \right) . \end{aligned}$$

#### Proof

Estimate (4.4a) follows from the well-known asymptotic expansion of the modified Bessel function for large argument (see, [27, (86)] with reference to [30, p. 270]). For (4.4b), we use the asymptotic expansion from [14, 10.40.10,.11,.12]

$$ \operatorname {e}\nolimits ^{z}\left( K_{\mu }\left( z\right) -K_{\mu +1}\left( z\right) \right) =\left( \frac{\pi }{2z}\right) ^{\frac{1}{2} }\left( 1+R_{1}\left( \mu ,z\right) -\left( 1+R_{1}\left( \mu +1,z\right) \right) \right) $$

with^4^

$$ \left| R_{1}\left( \nu ,z\right) \right| \le \frac{\pi }{4}\left| \frac{4\nu ^{2}-1}{z}\right| \exp \left( \pi \frac{\left| \nu ^{2} -\tfrac{1}{4}\right| }{\left| z\right| }\right) . $$

Hence,

$$ \left| \operatorname {e}\nolimits ^{z}\left( K_{\mu }\left( z\right) -K_{\mu +1}\left( z\right) \right) \right| \le \left( \frac{\pi }{2\left| z\right| }\right) ^{\frac{1}{2}}\left( \left| R_{1}\left( \mu ,z\right) \right| +\left| R_{1}\left( \mu +1,z\right) \right| \right) \le \frac{C_{\mu }}{\left| z\right| ^{3/2}}\exp \left( \frac{C_{\mu }}{\left| z\right| }\right) . $$

Next we prove (4.4c) and start with some preparations. Recall the *n*-th harmonic number

$$ H_{n}:=\sum _{m=1}^{n}\frac{1}{m} $$

and the Euler–Mascheroni constant $\gamma =\lim _{n\rightarrow +\infty }\left( H_{n}-\ln n\right) =0.57721\;56649\;01532\;86060\;\dots$. For $\nu \in \mathbb {N}$, the combination of [14, 10.31.1] with [14, 10.25.2] leads to

4.5a $$\begin{aligned} K_{\mu }\left( z\right) =\left( \dfrac{z}{2}\right) ^{-\mu }\sum _{k=0}^{\infty }c_{\mu ,k}\left( z\right) \left( \dfrac{z}{2}\right) ^{2k} \end{aligned}$$

for

4.5b $$\begin{aligned} c_{\mu ,k}\left( z\right) :=\left\{ \begin{array}{ll} \dfrac{\left( -1\right) ^{k}}{2}\dfrac{(\mu -1-k)!}{k!}, &  k\in \left\{ 0,1,\ldots ,\mu -1\right\} ,\\ \left( -\ln \dfrac{z}{2}-\gamma +\frac{H_{k-\mu }+H_{k}}{2}\right) \frac{\left( -1\right) ^{\mu }}{\left( k-\mu \right) !k!}, &  k\in \left\{ \mu ,\mu +1,\ldots \right\} . \end{array} \right. \end{aligned}$$

First, we derive bounds for the function $K_{\mu }$ with $\mu \in \mathbb {N}_{0}$. From (4.5), we derive

4.6 $$\begin{aligned} K_{\mu }\left( z\right) =\left( \dfrac{z}{2}\right) ^{-\mu }\chi _{\mu }^{\operatorname {I}}\left( z\right) -\left( \gamma +\ln \dfrac{z}{2}\right) \left( \dfrac{z}{2}\right) ^{\mu }\chi _{\mu }^{\operatorname {II}}\left( z\right) +\left( \dfrac{z}{2}\right) ^{\mu }\chi _{\mu }^{\operatorname {III} }\left( z\right) \end{aligned}$$

with entire functions $\chi _{\mu }^{\operatorname {I}}$, $\chi _{\mu }^{\operatorname {II}}$, $\chi _{\mu }^{\operatorname {III}}$. Hence, there exists a constant $\tilde{C}_{\mu }\left( R\right)$ such that for any $z\in B_{R}$, it holds

$$ \left| \chi _{\mu }^{\operatorname {I}}\left( z\right) \right| +\left| \chi _{\mu }^{\operatorname {II}}\left( z\right) \right| +\left| \chi _{\mu }^{\operatorname {III}}\left( z\right) \right| \le \tilde{C}_{\mu }\left( R\right) . $$

For the estimate of $K_{\mu }$, an inequality of the complex logarithm is needed. We write $z\in \mathbb {C}$ in polar coordinates $z=r\operatorname {e} ^{\operatorname {i}\varphi }$, $r\in \mathbb {R}_{>0}$ and $\varphi \in ] -\pi ,\pi ]$, and get

$$ \ln z=\ln r+\operatorname {i}\varphi \quad {\text {so that}}\quad \left| \ln z\right| \le \left| \ln r\right| +\pi . $$

This leads to the estimate of $K_{\mu }$ for any $z\in B_{R}\backslash \left[ -R,0\right] :$

$$ \left| K_{\mu }\left( z\right) \right| \le \left( \left| \dfrac{z}{2}\right| ^{-\mu }+\left( \gamma +\pi +\left| \ln \left| \frac{z}{2}\right| \right| \right) \left| \dfrac{z}{2}\right| ^{\mu }+\left| \dfrac{z}{2}\right| ^{\mu }\right) \tilde{C}_{\mu }\left( R\right) . $$

In this way, the function $K_{\mu }$ can be estimated, first for μ=0, by

$$ \left| K_{0}\left( z\right) \right| \le C_{0}\left( R\right) \left( 1+\left| \ln \left| \frac{z}{2}\right| \right| \right) \quad \forall z\in B_{R}\backslash \left[ -R,0\right] . $$

For $\mu \in \left\{ 1,2,\ldots \right\}$, we use the fact that powers decay faster than any logarithmic growth to obtain

$$ \left( \gamma +\pi +\left| \ln \left| \frac{z}{2}\right| \right| \right) \left| \dfrac{z}{2}\right| ^{\mu }\le \hat{C}_{\mu }\left( R\right) \quad \forall z\in B_{R}\backslash \left[ -R,0\right] $$

for some constant $\hat{C}_{\mu }\left( R\right)$ and obtain

4.7 $$\begin{aligned} \left| K_{\mu }\left( z\right) \right| \le C_{\mu }\left( R\right) \left( \dfrac{\left| z\right| }{2}\right) ^{-\mu }\quad \forall z\in \overset{\bullet }{\mathbb {C}}_{\ge 0}\cap B_{R}\backslash \left[ -R,0\right] . \end{aligned}$$

For $z\in B_{R}$, the modulus of the prefactor $\operatorname {e}^{z}$ in (4.4c) is bounded by $\operatorname {e}^{R}$ so that (4.4c) follows.

Next we prove (4.4c) for half integers $\mu \in \left\{ -\frac{1}{2},\frac{1}{2},\frac{3}{2}\ldots \right\}$. The modified Bessel functions of half integer have a finite representation (see, e.g. [20, Chap. 2, (5)]):

$$ \operatorname {e}\nolimits ^{z}K_{\mu }\left( z\right) =\sqrt{\frac{\pi }{2} }\frac{\theta _{\mu -1/2}\left( z\right) }{z^{\mu }} $$

with the reverse Bessel polynomials $\theta _{n}$ (see, e.g. [14, 18.34.2], [20, Chap. 2, (5)]). Since $\theta _{\mu -1/2}$ is a polynomial of degree μ-1/2, estimate (4.4c) also holds for half integers with an adjusted constant $C_{\mu }\left( R\right)$ still depending only on μ and *R*. □

By considering (4.4a), (4.4b) for $\left| z\right| \ge 1$ and choosing R=1 in (4.4c) we obtain the following corollary.

#### Corollary 4.4

The modified Bessel function $K_{\mu }$ satisfies

4.8 $$\begin{aligned} \left| \operatorname {e}\nolimits ^{z}K_{\mu }\left( z\right) \right| \le C_{\mu }M_{\mu }\left( \left| z\right| \right) \qquad \forall z\in \mathbb {C}\backslash \mathbb {R}_{\le 0}, \end{aligned}$$

with $M_{\mu }$ as in (4.1a).

Let $$\mu \in \mathbb {Z}_{\ge 0}^{\operatorname {half}}$$. Then

4.9 $$\begin{aligned} \left| \operatorname {e}\nolimits ^{z}\left( K_{\mu }\left( z\right) -K_{\mu +1}\left( z\right) \right) \right| \le C_{\mu }\frac{N_{\mu }\left( \left| z\right| \right) }{\left| z\right| } \quad \forall z\in \mathbb {C}\backslash \mathbb {R}_{\le 0}, \end{aligned}$$

where the majorant $N_{\mu }:\mathbb {R}_{>0}\rightarrow \mathbb {R}_{>0}$ is given by

$$ N_{\mu }\left( r\right) :=\left\{ \begin{array}{ll} r^{-1/2} &  {\text {for }}r\ge 1,\\ r^{-\mu } &  {\text {for }}0<r\le 1. \end{array} \right. $$

The functions $M_{\mu }$, $N_{\mu }$ are strictly decreasing on $\mathbb {R} _{>0}$ and it holds

4.10 $$\begin{aligned} N_{\mu }\left( r\right) \le M_{\mu }\left( r\right) \quad \forall r\in \mathbb {R}_{>0}. \end{aligned}$$

#### Proof

The estimates are direct consequences of Lemma 4.3 by considering (4.4a), (4.4b) for $\left| z\right| \ge 1$ and choosing R=1 in (4.4c). The estimate of $\left| \operatorname {e}\nolimits ^{z}\left( K_{\mu }\left( z\right) -K_{\mu +1}\left( z\right) \right) \right|$ for $\left| z\right| \le 1$ follows by a triangle inequality

$$ \left| \operatorname {e}\nolimits ^{z}\left( K_{\mu }\left( z\right) -K_{\mu +1}\left( z\right) \right) \right| \le \left| \operatorname {e}\nolimits ^{z}K_{\mu }\left( z\right) \right| +\left| \operatorname {e}\nolimits ^{z}K_{\mu +1}\left( z\right) \right| $$

in combination with (4.4c). □

### Holomorphic norm extension

The functions in (3.2) depend on the Euclidean norms $\left\| \textbf{x}-\textbf{y}\right\|$ and $\left\| \textbf{x}-\textbf{Ry} \right\|$; to prove that they are κ-slowly varying requires to study holomorphic norm extensions and some preliminaries.

The bilinear form $\left\langle \cdot ,\cdot \right\rangle :\mathbb {C}^{d} \times \mathbb {C}^{d}\rightarrow \mathbb {C}$ is given, for $\textbf{u}=\left( u_{j}\right) _{j=1}^{d}$, $\textbf{v}=\left( v_{j}\right) _{j=1}^{d}$, by $\left\langle \textbf{u},\textbf{v}\right\rangle =\sum _{j=1}^{d}u_{j}v_{j}$. For $z\in \overset{\bullet }{\mathbb {C}}_{\ge 0}$, we choose $\arg z\in \left[ -\frac{\pi }{2},\frac{\pi }{2}\right]$ so that $z=\left| z\right| \operatorname {e}^{\operatorname {i}\arg z}$ and recall the definition of the principal square root:

$$ \sqrt{z}:=\sqrt{\left| z\right| }\operatorname {e}\nolimits ^{\left( \operatorname {i}\arg z\right) /2}. $$

This directly implies the relations

4.11 $$\begin{aligned} \left| \sqrt{z}\right| =\sqrt{\left| z\right| }\quad {\text {and}}\quad \operatorname {Re}\sqrt{z}=\sqrt{\left| z\right| } \cos \left( \frac{\arg z}{2}\right) . \end{aligned}$$

For $\operatorname {Re}z>0$, we can estimate $\operatorname {Re}\sqrt{z}$ from below by^5^

4.12 $$\begin{aligned} \operatorname {Re}\sqrt{z}\ge \sqrt{\left| z\right| }\left( 1-\frac{1}{2}\left( \frac{\arg z}{2}\right) ^{2}\right) \ge \sqrt{\left| z\right| }\left( 1-\frac{1}{8}\left( \frac{\operatorname {Im} z}{\operatorname {Re}z}\right) ^{2}\right) . \end{aligned}$$

For the imaginary part, we get for $\operatorname {Re}z>0:$

4.13 $$\begin{aligned} \left| \operatorname {Im}\sqrt{z}\right| =\sqrt{\left| z\right| }\left| \sin \left( \frac{\arg z}{2}\right) \right| \le \sqrt{\left| z\right| }\frac{\left| \arg z\right| }{2} \le \frac{\left| \operatorname {Im}z\right| }{2\operatorname {Re}z} \sqrt{\left| z\right| }. \end{aligned}$$

In the following, we will frequently use the elementary relation

4.14 $$\begin{aligned} z^{2}-\left| z\right| ^{2}=2\operatorname {i}z\operatorname {Im} z\quad \forall z\in \mathbb {C}{\text {.}} \end{aligned}$$

#### Lemma 4.5

Let $\left[ \textbf{a},\textbf{b}\right] \times \left[ \textbf{c},\textbf{d}\right] \subset \mathbb {R}^{d}\times \mathbb {R}^{d}$ be a block of axes-parallel boxes which are η-admissible (cf. (3.8)). Let

$$ \kappa \in ] 0,\kappa _{0}[ \quad {\text {with}}\quad \kappa _{0}:=1/4 $$

and let the semi-axes sums be defined by (3.9). Then, for $\left( \textbf{x},\textbf{y}\right) \in \overrightarrow{\mathcal {E} }_{\textbf{a},\textbf{b},\textbf{c},\textbf{d}}\left( \kappa \right)$ there holds $\operatorname {Re}\left\langle \textbf{x}-\textbf{y},\textbf{x} -\textbf{y}\right\rangle >0$ and the analytic extension of *r* in (3.1) to $\overrightarrow{\mathcal {E}}_{\textbf{a},\textbf{b},\textbf{c},\textbf{d} }\left( \kappa \right)$, given by

4.15 $$\begin{aligned} r\left( \textbf{x},\textbf{y}\right) :=\sqrt{\left\langle \textbf{x} -\textbf{y},\textbf{x}-\textbf{y}\right\rangle }\quad \forall \left( \textbf{x},\textbf{y}\right) \in \overrightarrow{\mathcal {E}}_{\textbf{a} ,\textbf{b},\textbf{c},\textbf{d}}\left( \kappa \right) , \end{aligned}$$

is well defined. The extension satisfies the estimates

4.16 $$\begin{aligned} \left| r\left( \textbf{x},\textbf{y}\right) \right| \le \left\| \textbf{x}-\textbf{y}\right\| \quad {\text {and}}\quad \operatorname {Re}r\left( \textbf{x},\textbf{y}\right) \ge \left( 1-12\kappa ^{2}\right) \left\| \textbf{x}-\textbf{y}\right\| \quad \forall \left( \textbf{x},\textbf{y} \right) \in \overrightarrow{\mathcal {E}}_{\textbf{a},\textbf{b},\textbf{c} ,\textbf{d}}\left( \kappa \right) . \end{aligned}$$

For the imaginary part, it holds

4.17 $$\begin{aligned} \left| \operatorname {Im}r\left( \textbf{x},\textbf{y}\right) \right| \le 4\kappa \left\| \textbf{x}-\textbf{y}\right\| \quad \forall \left( \textbf{x},\textbf{y}\right) \in \overrightarrow{\mathcal {E}}_{\textbf{a},\textbf{b},\textbf{c},\textbf{d} }\left( \kappa \right) . \end{aligned}$$

#### Remark 4.6

In the following estimates, we work out the dominant dependence with respect to κ; the prefactors are often estimated in a generous way in order to reduce technicalities in the notation, e.g. for $\kappa \in ] 0,\kappa _{0}[$ we estimate $\frac{1}{1-\kappa }\le \frac{4}{3}\le 2$. However, we emphasize that our estimates are always strict for the full range of parameters and not only valid “up to higher-order terms”.

#### Proof of Lemma 4.5

The upper bound in (4.16) follows by a Cauchy–Schwarz inequality and the first relation in (4.11).

Next we prove the lower bound in (4.16). Note that $\operatorname {Re} r\left( \textbf{x},\textbf{y}\right) >0$ will be shown at the end of this proof. We consider exemplarily the case that the last side $\left[ a_{d},b_{d}\right]$ of $\left[ \textbf{a},\textbf{b}\right]$ is extended to $\mathcal {E}_{a_{d},b_{d}}\left( \kappa \right)$. Let $\left( \textbf{x},\textbf{y}\right) \in \overrightarrow{\mathcal {E}}_{\textbf{a},\textbf{b}}^{d}\left( \kappa \right) \times \left[ \textbf{c},\textbf{d} \right]$. Choose $\xi _{d}\in \left[ a_{d},b_{d}\right]$ such that

4.18 $$\begin{aligned} \left| x_{d}-\xi _{d}\right| =\operatorname {dist}\left( x_{d},\left[ a_{d},b_{d}\right] \right) \quad {\text {and set}}\quad \textbf{x} _{\operatorname {R}}:=\left( \textbf{x}^{\prime },\xi _{d}\right) \in \left[ \textbf{a},\textbf{b}\right] . \end{aligned}$$

We will use the shorthands

4.19 $$\begin{aligned} \mathfrak {D}:=\max \left\{ \operatorname {diam}\left[ \textbf{a} ,\textbf{b}\right] ,\operatorname {diam}\left[ \textbf{c},\textbf{d}\right] \right\} \quad {\text {and}}\quad \delta :=\operatorname {dist}\left( \left[ \textbf{a},\textbf{b}\right] ,\left[ \textbf{c},\textbf{d}\right] \right) \end{aligned}$$

and conclude from (3.5) that

4.20 $$\begin{aligned} \left| \xi _{d}-x_{d}\right|&\le \max \left\{ \overline{b_{d} },\overline{a_{d}}-\frac{b_{d}-a_{d}}{2}\right\} =\frac{\rho _{d}^{2}-\left( \frac{b_{d}-a_{d}}{2}\right) ^{2}}{2\rho _{d}}\nonumber \\&\overset{{\text {(3.9)}}}{=}\frac{\kappa }{\eta }\left( b_{d}-a_{d}\right) \frac{\eta +\kappa }{\eta +2\kappa }\le \frac{\kappa }{\eta }\left( b_{d}-a_{d}\right) \nonumber \\&\le \frac{\kappa }{\eta }\mathfrak {D}\le \kappa \delta . \end{aligned}$$

From a reverse triangle inequality, it follows

4.21 $$\begin{aligned} \left\| \textbf{x}-\textbf{y}\right\| \ge \left\| \textbf{x} _{\operatorname {R}}-\textbf{y}\right\| -\left\| \textbf{x} _{\operatorname {R}}-\textbf{x}\right\| \ge \delta -\left| \xi _{d} -x_{d}\right| \end{aligned}$$

and the combination with (4.20) leads to

4.22 $$\begin{aligned} \left\| \textbf{x}-\textbf{y}\right\| \ge \left( 1-\kappa \right) \delta . \end{aligned}$$

This implies for $\left| \xi _{d}-x_{d}\right|$ the estimate

4.23 $$\begin{aligned} \left| \xi _{d}-x_{d}\right| \le \frac{\kappa }{1-\kappa }\left\| \textbf{x}-\textbf{y}\right\| \le 2\kappa \left\| \textbf{x} -\textbf{y}\right\| . \end{aligned}$$

Next, we investigate the real and imaginary part of $\left\langle \textbf{x}-\textbf{y},\textbf{x}-\textbf{y}\right\rangle$. We set $\omega :=\left\| \textbf{x}^{\prime }-\textbf{y}^{\prime }\right\|$ and $z_{d}:=x_{d}-y_{d}$. Note that ${\textbf{x}}^{\prime }$, ${\textbf{y} }^{\prime }\in {\mathbb {R}}^{d-1}$. We obtain the relations

4.24a $$\begin{aligned} \operatorname {Re}\left\langle \textbf{x}-\textbf{y},\textbf{x}-\textbf{y} \right\rangle&=\omega ^{2}+\operatorname {Re}\left( z_{d}^{2}\right) \overset{\text {(4.14)}}{=}\left\| \textbf{x}-\textbf{y} \right\| ^{2}-2\left( \operatorname {Im}z_{d}\right) ^{2}, \end{aligned}$$

4.24b $$\begin{aligned} \operatorname {Im}\left\langle \textbf{x}-\textbf{y},\textbf{x}-\textbf{y} \right\rangle&\overset{\text {(4.14)}}{=}2\operatorname {Re} z_{d}\operatorname {Im}z_{d}. \end{aligned}$$

For the imaginary and real part of $z_{d}$, it holds

4.25 $$\begin{aligned} \left| \operatorname {Im}z_{d}\right|&=\left| \operatorname {Im} \left( x_{d}-\xi _{d}\right) \right| \le \left| \xi _{d} -x_{d}\right| \le 2\kappa \left\| \textbf{x}-\textbf{y}\right\| , \end{aligned}$$

4.26 $$\begin{aligned} \left| \operatorname {Re}z_{d}\right|&\le \left\| \textbf{x} -\textbf{y}\right\| , \end{aligned}$$

so that

4.27a $$\begin{aligned} \operatorname {Re}\left\langle \textbf{x}-\textbf{y},\textbf{x}-\textbf{y} \right\rangle&\ge \left( 1-8\kappa ^{2}\right) \left\| \textbf{x} -\textbf{y}\right\| ^{2}\overset{\kappa <1/4}{\ge }\frac{\left\| \textbf{x}-\textbf{y}\right\| ^{2}}{2}, \end{aligned}$$

4.27b $$\begin{aligned} \left| \operatorname {Im}\left\langle \textbf{x}-\textbf{y},\textbf{x} -\textbf{y}\right\rangle \right|&\le 4\kappa \left\| \textbf{x} -\textbf{y}\right\| ^{2} \end{aligned}$$

follow. Note that the condition $\kappa \in ] 0,\kappa _{0}[$ ensures that $\operatorname {Re}\left\langle \textbf{x}-\textbf{y},\textbf{x}-\textbf{y}\right\rangle >0$, and the function *r* is well defined by (4.15). Hence, we may apply (4.27) in (4.12) to estimate

4.28 $$\begin{aligned} \operatorname {Re}\sqrt{\left\langle \textbf{x}-\textbf{y},\textbf{x} -\textbf{y}\right\rangle }&\ge \left( 1-8\kappa ^{2}\right) \sqrt{\left| \left\langle \textbf{x}-\textbf{y},\textbf{x}-\textbf{y} \right\rangle \right| }\nonumber \\&\ge \left( 1-8\kappa ^{2}\right) \sqrt{\operatorname {Re}\left\langle \textbf{x}-\textbf{y},\textbf{x}-\textbf{y}\right\rangle }\nonumber \\&\ge \left( 1-8\kappa ^{2}\right) ^{3/2}\left\| \textbf{x} -\textbf{y}\right\| . \end{aligned}$$

Again, $\kappa \in ] 0,\kappa _{0}[$ implies that the κ-dependent factor in the right-hand side of (4.28) is positive. A straightforward calculation shows that

$$ \left( 1-8\kappa ^{2}\right) ^{3/2}\ge 1-12\kappa ^{2}\quad \forall \kappa \in ] 0,\kappa _{0}[ \text {.} $$

For the imaginary part of *r* we employ (4.13), the first inequality in (4.16), (4.27a) and (4.27b) to get

$$ \left| \operatorname {Im}\sqrt{\left\langle \textbf{x}-\textbf{y},\textbf{x}-\textbf{y}\right\rangle }\right| \le 4\kappa \left\| \textbf{x}-\textbf{y}\right\| . $$

□

#### Lemma 4.7

Let $\left[ \textbf{a},\textbf{b}\right] \times \left[ \textbf{c},\textbf{d}\right] \subset \mathbb {R}^{d}\times \mathbb {R}^{d}$ be a block of axes-parallel boxes which satisfy (3.8). Let $\kappa \in ] 0,\kappa _{1}[$ for $\kappa _{1}:=1/6$, and let the semi-axes sums be defined by (3.9). Then,

1. The holomorphic extension of the norm *r* to $\overrightarrow{\mathcal {E}}_{\textbf{a},\textbf{b},\textbf{c},\textbf{d} }\left( \kappa \right)$ can be estimated by:
  4.29 $$\begin{aligned} \left. \begin{array}{rl} \left| r\left( \textbf{x},\textbf{y}\right) \right| \le &  \left( 1+\kappa +2\eta \right) \operatorname {dist}\left( \left[ \textbf{a} ,\textbf{b}\right] ,\left[ \textbf{c},\textbf{d}\right] \right) \\ &  \\ \operatorname {Re}r\left( \textbf{x},\textbf{y}\right) \ge &  \left( 1-4\kappa \right) \operatorname {dist}\left( \left[ \textbf{a},\textbf{b} \right] ,\left[ \textbf{c},\textbf{d}\right] \right) \end{array} \right\} \quad \forall \left( \textbf{x},\textbf{y}\right) \in \overrightarrow{\mathcal {E}}_{\textbf{a},\textbf{b},\textbf{c},\textbf{d} }\left( \kappa \right) . \end{aligned}$$
2. Assume in addition that $\left[ \textbf{a},\textbf{b}\right] \times \left[ \textbf{c},\textbf{d}\right] \subset H_{+}\times H_{-}$. The function $r_{+}$ (cf. (2.3)) can be estimated for any $\kappa \in ] 0,\min \left\{ \kappa _{1},\frac{1}{3\beta }\right\} [$ by
  4.30 $$\begin{aligned} \left. \begin{array}{rl} \left| r_{+}\left( \textbf{x},\textbf{y}\right) \right| \le &  \left( 1+4\beta \right) \left| r\left( \textbf{x},\textbf{y}\right) \right| \\ &  \\ \operatorname {Re}r_{+}\left( \textbf{x},\textbf{y}\right) \ge &  \left( 1-3\beta \kappa \right) \operatorname {Re}r\left( \textbf{x},\textbf{y}\right) \end{array} \right\} \quad \forall \left( \textbf{x},\textbf{y}\right) \in \overrightarrow{\mathcal {E}}_{\textbf{a},\textbf{b},\textbf{c},\textbf{d} }\left( \kappa \right) . \end{aligned}$$

*Proof of (4.29)* First, we derive estimates for $\left\| \textbf{x}-\textbf{y}\right\|$. Again, we only consider the case that the last interval $\left[ a_{d},b_{d}\right]$ in $\left[ \textbf{a},\textbf{b}\right]$ is extended to $\mathcal {E}_{a_{d},b_{d}}\left( \kappa \right)$. Let $\left( \textbf{x},\textbf{y}\right) \in \overrightarrow{\mathcal {E}}_{\textbf{a},\textbf{b}}^{d}\left( \kappa \right) \times \left[ \textbf{c},\textbf{d} \right]$ and $\mathfrak {D}$, δ as in (4.19). Let $\xi _{d} \in \left[ a_{d},b_{d}\right]$ and $\textbf{x}_{\operatorname {R}}\in \left[ \textbf{a},\textbf{b}\right]$ be as in the previous proof, see (4.18). The lower estimate follows by combining (4.22) with (4.16):

$$ \operatorname {Re}r\left( \textbf{x},\textbf{y}\right) \ge \left( 1-12\kappa ^{2}\right) \left( 1-\kappa \right) \delta \ge \left( 1-4\kappa \right) \delta \quad \forall \kappa \in ] 0,\kappa _{0}[. $$

The upper estimate is implied by the first inequality in (4.16) and

4.31 $$\begin{aligned} \left\| \textbf{x}-\textbf{y}\right\|&\le \left\| \textbf{x} _{\operatorname {R}}-\textbf{y}\right\| +\left\| \textbf{x} _{\operatorname {R}}-\textbf{x}\right\| \le \delta +2\mathfrak {D}+\left| \xi _{d}-x_{d}\right| \nonumber \\&\le \delta +2\mathfrak {D}+\kappa \delta \le \left( 1+2\eta +\kappa \right) \delta , \end{aligned}$$

where we have used (4.20) in the penultimate last step. □

*Proof of (4.30)* To estimate the function $r_{+}$, we consider first the analytic extension for each of the first $\left( d-1\right)$ coordinates. Note that $\left[ \textbf{a},\textbf{b}\right] \times \left[ \textbf{c},\textbf{d}\right] \subset H_{+}\times H_{-}$ implies $x_{d}-y_{d}>0$. It is straightforward to obtain from this and β>0:

4.32 $$\begin{aligned} \operatorname {Re}r_{+}\left( \textbf{x},\textbf{y}\right) \ge \operatorname {Re}r\left( \textbf{x},\textbf{y}\right) \ge \left( 1-3\beta \kappa \right) \operatorname {Re}r\left( \textbf{x},\textbf{y}\right) . \end{aligned}$$

An upper estimate follows from

4.33 $$\begin{aligned} \left| r_{+}\left( \textbf{x},\textbf{y}\right) \right|&\le \left| r\left( \textbf{x},\textbf{y}\right) \right| +\beta \left\| \textbf{x}-\textbf{y}\right\| \nonumber \\&\overset{\text {(4.16)}}{\le }\left| r\left( \textbf{x} ,\textbf{y}\right) \right| +\frac{\beta }{1-12\kappa ^{2}}\operatorname {Re} r\left( \textbf{x},\textbf{y}\right) \le \left( 1+4\beta \right) \left| r\left( \textbf{x},\textbf{y}\right) \right| . \end{aligned}$$

Next, we consider the case that the last interval $\left[ a_{d},b_{d}\right]$ in $\left[ \textbf{a},\textbf{b}\right]$ is extended to $\mathcal {E} _{a_{d},b_{d}}\left( \kappa \right)$ and sketch afterwards the case that $\left[ c_{d},d_{d}\right]$ in $\left[ \textbf{c},\textbf{d}\right]$ is extended to $\mathcal {E}_{c_{d},d_{d}}\left( \kappa \right)$. Let $\left( \textbf{x},\textbf{y}\right) \in \overrightarrow{\mathcal {E}}_{\textbf{a},\textbf{b}}^{d}\left( \kappa \right) \times \left[ \textbf{c},\textbf{d} \right]$. For j=d, the splitting

$$ x_{d}-y_{d}=\xi _{d}-y_{d}+x_{d}-\xi _{d} $$

with $\xi _{d}$ as in (4.18) is employed. By using β>0, $\xi _{d}-y_{d}>0$, (4.22), and (4.20) we obtain

4.34 $$\begin{aligned} \operatorname {Re}r_{+}\left( \textbf{x},\textbf{y}\right)&\ge \operatorname {Re}r\left( \textbf{x,y}\right) +\beta \left( \xi _{d} -y_{d}\right) -\beta \left| x_{d}-\xi _{d}\right| \nonumber \\&\ge \operatorname {Re}r\left( \textbf{x,y}\right) -\beta \left| x_{d}-\xi _{d}\right| \nonumber \\&\ge \operatorname {Re}r\left( \textbf{x,y}\right) -\beta \kappa \delta . \end{aligned}$$

The combination with the second estimate in (4.29) yields

4.35 $$\begin{aligned} \operatorname {Re}r_{+}\left( \textbf{x},\textbf{y}\right) \ge \left( 1-3\beta \kappa \right) \operatorname {Re}r\left( \textbf{x},\textbf{y}\right) . \end{aligned}$$

Hence, the lower bound in (4.30) follows from (4.32) and (4.35). The upper bound follows as for the extension of the first $\left( d-1\right)$ coordinates.

Finally we sketch the proof for the case that $\left[ c_{d},d_{d}\right]$ in $\left[ \textbf{c},\textbf{d}\right]$ is extended to $\mathcal {E} _{c_{d},d_{d}}\left( \kappa \right)$. Let $\left( \textbf{x},\textbf{y} \right) \in \left[ \textbf{a},\textbf{b}\right] \times \overrightarrow{\mathcal {E}}_{\textbf{c},\textbf{d}}^{d}\left( \kappa \right)$. We choose $\zeta _{d}\in \left[ c_{d},d_{d}\right]$ such that $\left| y_{d}-\zeta _{d}\right|$ is minimal. As before, we obtain

$$ x_{d}-y_{d}=x_{d}-\zeta _{d}+\zeta _{d}-y_{d}\quad {\text {with}}\quad x_{d} -\zeta _{d}>0 $$

and, in turn:

$$\begin{aligned} \operatorname {Re}r_{+}\left( \textbf{x},\textbf{y}\right) \ge \operatorname {Re}r\left( \textbf{x,y}\right) +\beta \left( x_{d}-\zeta _{d}\right) -\beta \left| \zeta _{d}-y_{d}\right| \ge \operatorname {Re} r\left( \textbf{x,y}\right) -\beta \left| y_{d}-\zeta _{d}\right| \ge \operatorname {Re}r\left( \textbf{x,y}\right) -\beta \kappa \delta . \end{aligned}$$

The rest of the proof is a repetition of the arguments of the previous case. □

### Analysis of $\Theta _{\nu ,s}^{\operatorname {illu}}$ and $\Theta _{\nu ,s}^{\operatorname {refl}}$

The proof that $\Theta _{\nu ,s}^{\operatorname {illu}}$ is κ-slowly varying follows from the estimates of the Macdonald function and the holomorphic extension of the norm. The assertion for $\Theta _{\nu ,s}^{\operatorname {refl}}$ in addition employs a reflection argument.

#### Lemma 4.8

Let $0<\eta \le \eta _{0}$ be as in Definition 3.3 and $\kappa \in ] 0,\min \left\{ \frac{1}{6},\frac{1}{3\beta },\frac{\beta }{6}\right\} [$. The families of functions $\mathcal {F}_{\nu }^{\operatorname {illu}}$ and $\mathcal {F}_{\nu }^{\operatorname {refl}}$ as in (3.4) are κ-slowly varying: for any η-admissible block $B=\left[ \textbf{a},\textbf{b} \right] \times \left[ \textbf{c},\textbf{d}\right] \subset H_{+}\times H_{+}$ and $\delta :=\operatorname {dist}\left( \left[ \textbf{a},\textbf{b}\right] ,\left[ \textbf{c},\textbf{d}\right] \right)$, it holds:

4.36a $$\begin{aligned} \max _{\left( \textbf{x},\textbf{y}\right) \in \overrightarrow{\mathcal {E} }_{\textbf{a},\textbf{b},\textbf{c},\textbf{d}}\left( \kappa \right) }\left| \Theta _{\nu ,s}^{\operatorname {illu}}\left( \textbf{x} ,\textbf{y}\right) \right|&\le C_{\nu }\left( \frac{\left| s\right| }{\delta }\right) ^{\nu +1/2}M_{\nu +1/2}\left( \left| s\right| \delta \right) , \end{aligned}$$

4.36b $$\begin{aligned} \max _{\left( \textbf{x},\textbf{y}\right) \in \overrightarrow{\mathcal {E} }_{\textbf{a},\textbf{b},\textbf{c},\textbf{d}}\left( \kappa \right) }\left| \Theta _{\nu ,s}^{\operatorname {refl}}\left( \textbf{x} ,\textbf{y}\right) \right|&\le C_{\nu ,\beta }\left( \frac{\left| s\right| }{\delta }\right) ^{\nu +1/2}M_{\nu +1/2}\left( \left| s\right| \delta \right) \end{aligned}$$

with $M_{\nu +1/2}$ as in (4.1a).

The constants $C_{\mathcal {F}}$, τ, and reference functions λ in Definition 3.5 can be chosen as defined in Theorem 4.2. In particular, the constants are independent of κ.

#### Proof

First, the claim is proved for $\Theta _{\nu ,s}^{\operatorname {illu}}$ (considered as a function on $\mathbb {R}^{d}\times \mathbb {R}^{d}$). Let $B:=\left[ \textbf{a},\textbf{b}\right] \times \left[ \textbf{c},\textbf{d}\right] \subset \mathbb {R}^{d}\times \mathbb {R}^{d}$ be an η-admissible block. We identify $\left. \Theta _{\nu ,s}^{\operatorname {illu} }\right| _{B}$ with its holomorphic extension to $\overrightarrow{\mathcal {E}}_{\textbf{a},\textbf{b},\textbf{c},\textbf{d} }\left( \kappa \right)$ and consider exemplarily the holomorphic extension to $\overrightarrow{\mathcal {E}}_{\textbf{a},\textbf{b}}^{d}\left( \kappa \right) \times \left[ \textbf{c},\textbf{d}\right]$. For $\left( \textbf{x},\textbf{y}\right) \in \overrightarrow{\mathcal {E}}_{\textbf{a},\textbf{b}}^{d}\left( \kappa \right) \times \left[ \textbf{c},\textbf{d} \right]$ we write $\textbf{z}:=\textbf{x}-\textbf{y}$ and denote by $r:=r\left( \textbf{x},\textbf{y}\right)$ the holomorphic norm extension as in (4.15). We use the shorthands $\mathfrak {D},\delta$ as in (4.19). Then,

4.37 $$\begin{aligned} \max _{\left( \textbf{x},\textbf{y}\right) \in \overrightarrow{\mathcal {E} }_{\textbf{a},\textbf{b}}^{d}\left( \kappa \right) \times \left[ \textbf{c},\textbf{d}\right] }\left| \Theta _{\nu ,s}^{\operatorname {illu} }\left( \textbf{x},\textbf{y}\right) \right| =\frac{1}{\left( 2\pi \right) ^{\nu +3/2}}\max _{\left( \textbf{x},\textbf{y}\right) \in \overrightarrow{\mathcal {E}}_{\textbf{a},\textbf{b}}^{d}\left( \kappa \right) \times \left[ \textbf{c},\textbf{d}\right] }\left( \left| \frac{s}{r}\right| ^{\nu +1/2}\left| \operatorname {e}\nolimits ^{sr} K_{\nu +1/2}\left( sr\right) \right| \right) . \end{aligned}$$

From Lemma 4.7, we conclude that $\operatorname {Re}r>0$ for $\kappa \in ] 0,\frac{1}{6}[$, and for $s\in \overset{\bullet }{\mathbb {C}}_{\ge 0}$, we have

$$ sr\in \left\{ z\in \mathbb {C}\backslash \mathbb {R}_{\le 0}\mid \frac{\left| s\right| \delta }{3}\le \left| z\right| \le \left( \frac{7}{6}+2\eta _{0}\right) \left| s\right| \delta \right\} . $$

As a consequence,

$$ \max _{\left( \textbf{x},\textbf{y}\right) \in \overrightarrow{\mathcal {E} }_{\textbf{a},\textbf{b}}^{d}\left( \kappa \right) \times \left[ \textbf{c},\textbf{d}\right] }\left| \frac{s}{r}\right| ^{\nu +1/2}\le \max _{\left( \textbf{x},\textbf{y}\right) \in \overrightarrow{\mathcal {E}}_{\textbf{a},\textbf{b}}^{d}\left( \kappa \right) \times \left[ \textbf{c},\textbf{d}\right] }\left| \frac{3\,s}{\delta }\right| ^{\nu +1/2}. $$

For the second factor in (4.37), we employ Corollary 4.4, i.e.

$$ \max _{\left( \textbf{x},\textbf{y}\right) \in \overrightarrow{\mathcal {E} }_{\textbf{a},\textbf{b}}^{d}\left( \kappa \right) \times \left[ \textbf{c},\textbf{d}\right] }\left| \operatorname {e}\nolimits ^{sr} K_{\nu +1/2}\left( sr\right) \right| \le C_{\nu }M_{\nu +1/2}\left( \left| sr\right| \right) $$

and arrive (with an adjusted constant $C_{\nu }$) at

$$ \max _{\left( \textbf{x},\textbf{y}\right) \in \overrightarrow{\mathcal {E} }_{\textbf{a},\textbf{b}}^{d}\left( \kappa \right) \times \left[ \textbf{c},\textbf{d}\right] }\left| \Theta _{\nu ,s}^{\operatorname {illu} }\left( \textbf{x},\textbf{y}\right) \right| \le C_{\nu }\left( \frac{\left| s\right| }{\delta }\right) ^{\nu +1/2}M_{\nu +1/2}\left( \left| s\right| \delta \right) , $$

where $C_{\nu }$ only depends on ν.

To show that $\Theta _{\nu ,s}^{\operatorname {refl}}$ is κ-slowly varying, we define the reflected box

$$ \textbf{R}\left[ \textbf{c},\textbf{d}\right] :=\left\{ \textbf{Ry}:\textbf{y}\in \left[ \textbf{c,d}\right] \right\} \subset H_{-} $$

and set

$$ \textbf{z}_{-}:=\textbf{x}-\textbf{Ry,}{\quad }d_{-}:=\sqrt{\left\langle \textbf{z}_{-},\textbf{z}_{-}\right\rangle },\quad z_{d}^{-}:=x_{d}+y_{d}. $$

The η-admissibility of $\left[ \textbf{a},\textbf{b}\right] \times \left[ \textbf{c},\textbf{d}\right]$ implies the η-admissibility of $\left[ \textbf{a},\textbf{b}\right] \times \textbf{R}\left[ \textbf{c},\textbf{d}\right]$ as can be seen from the following reasoning: choose $\left( \textbf{x}_{0},\textbf{y}_{0}\right) \in \left[ \textbf{a},\textbf{b}\right] \times \left[ \textbf{c},\textbf{d}\right]$ such that

$$ \delta _{\operatorname {refl}}:=\operatorname {dist}\left( \left[ \textbf{a},\textbf{b}\right] ,\textbf{R}\left[ \textbf{c},\textbf{d}\right] \right) =\left\| \textbf{x}_{0}-\textbf{Ry}_{0}\right\| . $$

Then, $\delta =\operatorname {dist}\left( \left[ \textbf{a},\textbf{b}\right] ,\left[ \textbf{c},\textbf{d}\right] \right)$ can be estimated by using $\left| x_{0,d}-y_{0,d}\right| \le x_{0,d}+y_{0,d}$ for any $\textbf{x}_{0},\textbf{y}_{0}\in H_{+}:$

4.38 $$\begin{aligned} \delta \le \left\| \textbf{x}_{0}-\textbf{y}_{0}\right\| =\sqrt{\left\| \textbf{x}_{0}^{\prime }-\textbf{y}_{0}^{\prime }\right\| ^{2}+\left( x_{0,d}-y_{0,d}\right) ^{2}}\le \left\| \textbf{x} _{0}-\textbf{Ry}_{0}\right\| =\delta _{\operatorname {refl}} \end{aligned}$$

and the auxiliary statement follows from

4.39 $$\begin{aligned} \max \left\{ \operatorname {diam}\left[ \textbf{a},\textbf{b}\right] ,\operatorname {diam}\textbf{R}\left[ \textbf{c},\textbf{d}\right] \right\} =\max \left\{ \operatorname {diam}\left[ \textbf{a},\textbf{b}\right] ,\operatorname {diam}\left[ \textbf{c},\textbf{d}\right] \right\} \le \eta \delta \le \eta \delta _{\operatorname {refl}}. \end{aligned}$$

Recall that $\Theta _{\nu ,s}^{\operatorname {refl}}$ is defined in (3.2b) via the function $\sigma _{\nu }$ from (2.12) and we estimate the prefactor in (2.12) first. By the same arguments as for the second inequality in (4.30), it follows with $\tilde{\beta }=1/\beta$ and $\tilde{r}_{+}\left( \textbf{z}\right) :=r\left( \textbf{z}\right) +\tilde{\beta } z_{d}$

$$\begin{aligned} \operatorname {Re}\left( \beta d_{-}+z_{d}^{-}\right)&=\beta \operatorname {Re}\tilde{r}_{+}\left( \textbf{z}^{-}\right) \ge \left( \beta -3\kappa \right) \operatorname {Re}d_{-}\ge \frac{\beta }{2} \operatorname {Re}d_{-}\\&\overset{\text {(4.16)}}{\ge }\frac{\beta }{2}\left( 1-12\kappa ^{2}\right) \left\| \textbf{z}_{-}\right\| \overset{\kappa <1/6}{\ge }\frac{\beta }{3}\left\| \textbf{z}_{-}\right\| \qquad \forall \left( \textbf{x},\textbf{y}\right) \in \overrightarrow{\mathcal {E}}_{\textbf{a} ,\textbf{b},\textbf{c},\textbf{d}}\left( \kappa \right) . \end{aligned}$$

For the numerator in the prefactor of $\sigma _{\nu }$, it holds

$$ \left| z_{d}^{-}-\beta d_{-}\right| \le \left( 1+\beta \right) \left\| \textbf{z}_{-}\right\| $$

so that

$$ \max _{\left( \textbf{x},\textbf{y}\right) \in \overrightarrow{\mathcal {E} }_{\textbf{a},\textbf{b},\textbf{c},\textbf{d}}\left( \kappa \right) }\left| \frac{z_{d}^{-}-\beta d_{-}}{z_{d}^{-}+\beta d_{-}}\right| \le 3\frac{1+\beta }{\beta }. $$

The estimates for the function $\Theta _{\nu ,s}^{\operatorname {illu}}$ then imply:

$$\begin{aligned} \max _{\left( \textbf{x},\textbf{y}\right) \in \overrightarrow{\mathcal {E} }_{\textbf{a},\textbf{b}}^{d}\left( \kappa \right) \times \left[ \textbf{c},\textbf{d}\right] }\left| \Theta _{\nu ,s}^{\operatorname {refl} }\left( \textbf{x},\textbf{y}\right) \right|&=3\frac{1+\beta }{\beta }\max _{\left( \textbf{x},\textbf{y}\right) \in \overrightarrow{\mathcal {E} }_{\textbf{a},\textbf{b}}^{d}\left( \kappa \right) \times \textbf{R}\left[ \textbf{c},\textbf{d}\right] }\left| \Theta _{\nu ,s}^{\operatorname {illu} }\left( \textbf{x},\textbf{y}\right) \right| \\&\le C_{\nu ,\beta }\left( \frac{\left| s\right| }{\delta _{\operatorname {refl}}}\right) ^{\nu +1/2}M_{\nu +1/2}\left( \left| s\right| \delta _{\operatorname {refl}}\right) . \end{aligned}$$

Since $\delta \le \delta _{\operatorname {refl}}$ and $M_{\nu +1/2}$ is strictly decreasing, the assertion follows by adjusting $C_{\nu ,\beta }$. □

### Analysis of $\Theta _{\nu ,s}^{\operatorname {imp}}$

In this section, it will be shown that the family of functions $\mathcal {F} _{\nu }^{\operatorname {imp}}$ (see (3.4c)) is κ-slowly varying. We employ the definition (3.2c) and the definition of $\psi _{\nu ,s}$ as in (2.12)

4.40 $$\begin{aligned} \Theta _{\nu ,s}^{\operatorname {imp}}\left( \textbf{x},\textbf{y}\right) =-\frac{\beta }{\pi }\left( \frac{s^{2}}{2\pi }\right) ^{\nu +1/2}\frac{1}{s}\int \limits _{0}^{\infty }\operatorname {e}\nolimits ^{-sy}q_{\nu }\left( \textbf{z},y\right) dy \end{aligned}$$

and

$$ \textbf{z}:=\textbf{x}-\mathbf {Ry\quad }{\text {and}}\quad r:=\left\| \textbf{z}\right\| . $$

We start with an estimate of the function $q_{\nu }\left( \textbf{z},y\right)$ as in (2.13). We employ the following notation and setting: let $B=\left[ \textbf{a},\textbf{b}\right] \times \left[ \textbf{c},\textbf{d}\right] \subset H_{+}\times H_{+}$ be an η-admissible block and $\delta :=\operatorname {dist}\left( \left[ \textbf{a},\textbf{b}\right] ,\left[ \textbf{c},\textbf{d}\right] \right)$. For $\left( \textbf{x},\textbf{y}\right) \in \overrightarrow{\mathcal {E}}_{\textbf{a},\textbf{b},\textbf{c},\textbf{d}}\left( \kappa \right)$ let $\textbf{z}:=\textbf{x} -\textbf{Ry}$. The analytically extended functions (cf. Footnote 3) *r*, $r_{+}:\overrightarrow{\mathcal {E}}_{\textbf{a},\textbf{b},\textbf{c},\textbf{d} }\left( \kappa \right) \rightarrow \mathbb {C}$ are given by

$$ r\left( \textbf{z}\right) =\sqrt{\left\langle \textbf{z},\textbf{z} \right\rangle }\quad {\text {and}}\quad r_{+}\left( \textbf{z}\right) =r\left( \textbf{z}\right) +\beta z_{d}\text {.} $$

#### Remark 4.9

The analytic extension of *r* and $r_{+}$ induces analytic extensions of μ (in 2.6)):

$$ \mu \left( \textbf{z}^{\prime },t\right) :=r\left( \left( {\begin{array}{c}\textbf{z}^{\prime }\\ t\end{array}}\right) \right) \quad {\text {with}}\quad \textbf{z}^{\prime }\in \overrightarrow{\mathcal {E}}_{\textbf{a},\textbf{b},\textbf{c},\textbf{d} }^{\prime }\left( \kappa \right) :=\left\{ \left( \textbf{x}-\textbf{Ry} \right) ^{\prime }\mid \left( \textbf{x},\textbf{y}\right) \in \overrightarrow{\mathcal {E}}_{\textbf{a},\textbf{b},\textbf{c},\textbf{d} }\left( \kappa \right) \right\} $$

and $t\in [ z_{d},\infty [$ is related to the integration variable in (2.12). In turn, the analytic extension to $\overrightarrow{\mathcal {E}}_{\textbf{a},\textbf{b},\textbf{c},\textbf{d} }^{\prime }\left( \kappa \right)$ of *y* is induced by (2.5), of *t* by (2.7), and of $\tilde{\mu }$ by (2.10).

By introducing the notation

$$ \chi =a^{2}-\left( 1-\beta ^{2}\right) \omega ^{2}\quad {\text {with}}\quad a:=y+r_{+},\quad \omega ^{2}:=\left\langle \textbf{z}^{\prime },\textbf{z} ^{\prime }\right\rangle $$

a more explicit representation of the functions $\tilde{\mu }$ and *t* is given by:

4.41 $$\begin{aligned} \tilde{\mu }\left( \textbf{z},y\right) =\frac{a\sqrt{\chi }+\beta \omega ^{2} }{\beta a+\sqrt{\chi }}\quad {\text {and}}\quad t\left( \textbf{z},y\right) =\frac{a^{2}-\omega ^{2}}{\beta a+\sqrt{\chi }}, \end{aligned}$$

which can be verified by some straightforward manipulations.

Recall the definition of the function $\tilde{\mu }$ as in (2.10) and adopt the notation $\tilde{\mu }^{\prime }\left( \textbf{z},y\right) =\partial \tilde{\mu }\left( \textbf{z},y\right) /\partial y$. An explicit calculation yields $q_{\nu }=q_{\nu }^{\operatorname {I}}+q_{\nu } ^{\operatorname {II}}$ with

$$\begin{aligned} q_{\nu }^{\operatorname {I}}\left( \textbf{z},y\right)&:=s\left( \tilde{\mu }^{\prime }-\frac{\tilde{\mu }\left( \tilde{\mu }+\beta t\right) }{\left( t+\beta \tilde{\mu }\right) ^{2}}\right) \frac{\operatorname {e} \nolimits ^{s\tilde{\mu }}K_{\nu +1/2}\left( s\tilde{\mu }\right) }{\left( t+\beta \tilde{\mu }\right) \left( s\tilde{\mu }\right) ^{\nu +1/2}},\\ q_{\nu }^{\operatorname {II}}\left( \textbf{z},y\right)&:=s^{2}\tilde{\mu }^{\prime }\frac{\tilde{\mu }}{t+\beta \tilde{\mu }}\left( \frac{\operatorname {e}\nolimits ^{s\tilde{\mu }}\left( K_{\nu +1/2}\left( s\tilde{\mu }\right) -K_{\nu +3/2}\left( s\tilde{\mu }\right) \right) }{\left( s\tilde{\mu }\right) ^{\nu +1/2}}\right) . \end{aligned}$$

Clearly, a key role in the analysis of $q_{\nu }$ and, in turn, of $\Theta _{\nu ,s}^{\operatorname {imp}}$ is played by the function $\tilde{\mu }$, and two-sided estimates are stated in the following lemma.

#### Lemma 4.10

Let $\left[ \textbf{a},\textbf{b}\right] \times \left[ \textbf{c},\textbf{d}\right] \subset H_{+}\times H_{+}$ be a block of axes-parallel boxes that satisfy (3.8). There exist numbers $C_{\mathcal {E}}\ge 6$, $C_{0}>1$, and $c_{2}>0$ independent of all parameters and functions such that for any $\kappa \in ] 0,\frac{\beta ^{2} }{C_{\mathcal {E}}\left( 1+\beta \right) ^{3}}[$ and semi-axes sums defined by (3.9) and any $\left( \textbf{x},\textbf{y}\right) \in \overrightarrow{\mathcal {E}}_{\textbf{a},\textbf{b},\textbf{c},\textbf{d} }\left( \kappa \right)$ it holds for $\textbf{z}:=\textbf{x}-\textbf{Ry}$ and y≥0:

4.42a $$\begin{aligned} \left| \tilde{\mu }\left( \textbf{z},y\right) \right| \le C_{0} \frac{1+\beta }{\beta }\left( y+\left( 1+\beta \right) \left\| \textbf{z}\right\| \right) \end{aligned}$$

and

4.42b $$\begin{aligned} \operatorname {Re}\tilde{\mu }\left( \textbf{z},y\right) \ge C_{0}^{-1} \frac{\beta ^{2}}{\left( 1+\beta \right) ^{4}}\left( y+\left\| \textbf{z}\right\| \right) . \end{aligned}$$

For the sum $t+\beta \tilde{\mu }$, it holds

4.42c $$\begin{aligned} \operatorname {Re}\left( t\left( \textbf{z},y\right) +\beta \tilde{\mu }\left( \textbf{z},y\right) \right) \ge c_{2}\left( y+\beta \left\| \textbf{z}\right\| \right) \end{aligned}$$

and for the derivative:

4.42d $$\begin{aligned} \left| \frac{\partial \tilde{\mu }\left( \textbf{z},y\right) }{\partial y}\right| \le C_{0}\left( \frac{1+\beta }{\beta }\right) ^{2}. \end{aligned}$$

#### Remark 4.11

The upper bound for the range of κ in Lemma 4.10, i.e. $\frac{\beta ^{2}}{C_{\mathcal {E}}\left( 1+\beta \right) ^{3}}$, takes its maximal value $\frac{4}{27}C_{\mathcal {E} }^{-1}$ at β=2; hence, the upper bound for κ tends linearly to zero as $C_{\mathcal {E}}$ increases. It is also straightforward to verify that $C_{\mathcal {E}}\ge 6$ implies

$$ \frac{\beta ^{2}}{C_{\mathcal {E}}\left( 1+\beta \right) ^{3}}\le \frac{1}{6}\min \left\{ 1,\beta ^{-1}\right\} . $$

The proof of Lemma 4.10 is fairly technical and postponed to Sect. 5.

Lemma 4.10 allows us to estimate the function $q_{\nu }$.

#### Lemma 4.12

Let $0<\eta \le \eta _{0}$ be as in Definition 3.3. Let $\left[ \textbf{a},\textbf{b}\right] \times \left[ \textbf{c},\textbf{d}\right] \subset H_{+}\times H_{+}$ be a block of axes-parallel boxes that satisfy (3.8). Let $C_{\mathcal {E}},C_{0}>0$ be as in Lemma 4.10.

1. For any $\kappa \in ] 0,\frac{\beta ^{2}}{C_{\mathcal {E}}\left( 1+\beta \right) ^{3}}[$ and semi-axes sums defined by (3.9), any $\left( \textbf{x},\textbf{y}\right) \in \overrightarrow{\mathcal {E}}_{\textbf{a},\textbf{b},\textbf{c},\textbf{d} }\left( \kappa \right)$ and $\textbf{z}:=\textbf{x}-\textbf{Ry}$, the integral $\int \limits _{0}^{\infty }q_{\nu }\left( \textbf{z},y\right) dy$ exists as an improper Riemann integral.
2. The family of functions $\mathcal {F}_{\nu }^{\operatorname {imp}}$ in (3.4c) is κ-slowly varying: for any $\kappa \in ] 0,\frac{\beta ^{2}}{C_{\mathcal {E}}\left( 1+\beta \right) ^{3}}[$, any η-admissible block $B=\left[ \textbf{a},\textbf{b}\right] \times \left[ \textbf{c},\textbf{d}\right] \subset H_{+}\times H_{+}$, and with $\delta :=\operatorname {dist}\left( \left[ \textbf{a},\textbf{b}\right] ,\left[ \textbf{c},\textbf{d}\right] \right)$ it holds
  $$ \max _{\left( \textbf{x},\textbf{y}\right) \in \overrightarrow{\mathcal {E} }_{\textbf{a},\textbf{b},\textbf{c},\textbf{d}}\left( \boldsymbol{\rho } _{1},\boldsymbol{\rho } _{2}\right) }\left| \Theta _{\nu ,s}^{\operatorname {imp}}\left( \textbf{x},\textbf{y}\right) \right| \le C_{\nu ,\beta }\left( \frac{\left| s\right| }{\delta }\right) ^{\nu +1/2}W_{\nu +1/2}\left( \left| s\right| \delta \right) $$
  with $W_{\mu }$ as in (4.1b). The constants $C_{\mathcal {F}}$, τ, and reference function λ in Definition 3.5 can be chosen as defined in Theorem 4.2. In particular, the constants are independent of κ.

#### Proof

Let $\left( \textbf{x},\textbf{y}\right) \in \overrightarrow{\mathcal {E} }_{\textbf{a},\textbf{b},\textbf{c},\textbf{d}}\left( \kappa \right)$ and $\textbf{z}:=\textbf{x}-\textbf{Ry}$. We use the shorthands $r_{\operatorname {R}}=\left\| \textbf{z}\right\|$, $\tilde{\mu } =\tilde{\mu }\left( \textbf{z},y\right)$, and $\tilde{\mu }^{\prime }\left( \textbf{z},y\right) =\partial \tilde{\mu }\left( \textbf{z},y\right) /\partial y$. Remark 4.11 allows us to apply Lemmata 4.5 and 4.7, and the combination of (4.30) with (4.16) leads to

4.43 $$\begin{aligned} \operatorname {Re}r_{+}\left( \textbf{z}\right) \ge \left( 1-3\beta \kappa \right) \left( 1-12\kappa ^{2}\right) r_{\operatorname {R}}\ge \frac{1}{3}r_{\operatorname {R}}\quad {\text {and}}\quad \operatorname {Re}\left( r_{+}+y\right) \ge y+\frac{r_{\operatorname {R}}}{3} . \end{aligned}$$

Then, the inequalities (4.42) and relation (cf. (2.5)) $\tilde{\mu }+\beta t=y+r_{+}$ imply in view of Lemma 4.10

$$\begin{aligned} \left| \tilde{\mu }^{\prime }-\frac{\tilde{\mu }\left( \tilde{\mu }+\beta t\right) }{\left( t+\beta \tilde{\mu }\right) ^{2}}\right|&\le C_{0}\left( \frac{1+\beta }{\beta }\right) ^{2}+\frac{C_{0}}{c_{2}^{2}} \frac{1+\beta }{\beta }\left( \frac{y+\left( 1+\beta \right) r_{\operatorname {R}}}{y+\beta r_{\operatorname {R}}}\right) ^{2}\\&\le C_{0}\left( \frac{1+\beta }{\beta }\right) ^{2}+\frac{C_{0}}{c_{2} ^{2}}\frac{\left( 1+\beta \right) ^{3}}{\beta ^{3}}\le C_{0}^{\prime } \frac{\left( 1+\beta \right) ^{3}}{\beta ^{3}}. \end{aligned}$$

From Lemma 4.10 and $s\in \overset{\bullet }{\mathbb {C}}_{\ge 0}$, it follows (with $c_{0}^{\prime }:=C_{0}^{-1}\beta ^{2}\left( 1+\beta \right) ^{-4}$)

$$ s\tilde{\mu }\in \left\{ \zeta \in \mathbb {C}\backslash \mathbb {R}_{\le 0}\mid c_{0}^{\prime }\left| s\right| \left( y+r_{\operatorname {R}}\right) \le \left| \zeta \right| \le C_{0}\frac{1+\beta }{\beta }\left| s\right| \left( y+\left( 1+\beta \right) r_{\operatorname {R}}\right) \right\} . $$

This allows us to apply Corollary 4.4 and the monotonicity of $M_{\mu }$ so that

$$\begin{aligned} \left| q_{\nu }^{\operatorname {I}}\left( \textbf{z},y\right) \right|&\le C_{0}^{\prime }\frac{\left( 1+\beta \right) ^{3}}{\beta ^{3}}\left| s\right| \frac{M_{\nu +1/2}\left( c_{0}^{\prime }\left| s\right| \left( y+r_{\operatorname {R}}\right) \right) }{c_{2}\left( y+\beta r_{\operatorname {R}}\right) \left( c_{0}^{\prime }\left| s\right| \left( y+r_{\operatorname {R}}\right) \right) ^{\nu +1/2}}\\&\le C_{\nu ,\beta }\left| s\right| ^{2}\frac{M_{\nu +1/2}\left( c_{0}^{\prime }\left| s\right| \left( y+r_{\operatorname {R}}\right) \right) }{\left( \left| s\right| \left( y+r_{\operatorname {R} }\right) \right) ^{\nu +3/2}}. \end{aligned}$$

To estimate $q_{\nu }^{\operatorname {II}}$, we combine Lemma 4.10, Corollary 4.4, and (4.43) and obtain

$$ \left| q_{\nu }^{\operatorname {II}}\left( \textbf{z},y\right) \right| \le C_{\nu ,\beta }\left| s\right| ^{2}\frac{N_{\nu +1/2}\left( c_{0}^{\prime }\left| s\right| \left( y+r_{\operatorname {R}}\right) \right) }{\left( \left| s\right| \left( y+r_{\operatorname {R}}\right) \right) ^{\nu +3/2}}. $$

It follows that a majorant for the function $q_{\nu }\left( \textbf{z},\cdot \right)$ is given by a rational function without poles for $y\in ] 0,\infty [$ since $r_{R}>0$. For large *z*, we use that $M_{\nu +1/2}\left( z\right)$ as well as $N_{\nu +1/2}\left( z\right)$ decay with a speed of $\left| z\right| ^{-1/2}$ (cf. Corollary 4.4) so that $q_{\nu }\left( \textbf{z},\cdot \right)$ decays with a speed of $O\left( \left( y+r_{\operatorname {R}}\right) ^{-\nu -2}\right)$. From $\nu \ge -1/2$, we conclude that $\int \limits _{0}^{\infty }q_{\nu }\left( \textbf{z},y\right) dy$ exists as an improper Riemann integral.

For the estimate of the function $\Theta _{\nu ,s}^{\operatorname {imp}}$, we recall that $M_{\nu +1/2}$ is a majorant of $N_{\nu +1/2}$ (see (4.10)) so that

$$ \left| q_{\nu }\left( \textbf{z},y\right) \right| \le C_{\nu ,\beta }\left| s\right| ^{2}\frac{M_{\nu +1/2}\left( c_{0}^{\prime }\left| s\right| \left( y+r_{\operatorname {R}}\right) \right) }{\left( \left| s\right| \left( y+r_{\operatorname {R}}\right) \right) ^{\nu +3/2}}. $$

This leads to

4.44 $$\begin{aligned} \left| \Theta _{\nu ,s}^{\operatorname {imp}}\left( \textbf{x} ,\textbf{y}\right) \right| \le C_{\nu ,\beta }\left| s\right| ^{2\left( \nu +1\right) }\int \limits _{0}^{\infty }\frac{M_{\nu +1/2}\left( c_{0}^{\prime }\left| s\right| \left( y+r_{\operatorname {R}}\right) \right) }{\left( \left| s\right| \left( y+r_{\operatorname {R} }\right) \right) ^{\nu +3/2}}dy. \end{aligned}$$

In view of the piecewise definition of $M_{\mu }$ in (4.1a), we set

4.45 $$\begin{aligned} b=\max \left\{ 0,\frac{1}{c_{0}^{\prime }\left| s\right| } -r_{\operatorname {R}}\right\} \end{aligned}$$

and observe

$$ c_{0}^{\prime }\left| s\right| \left( b+r_{\operatorname {R}}\right) =\max \left\{ 1,c_{0}^{\prime }\left| s\right| r_{\operatorname {R} }\right\} . $$

This leads to the splitting $\left( \int \limits _{0}^{b}\ldots +\int \limits _{b}^{\infty }\ldots \right)$ of the integral (4.44). For the second summand, we get with an adjusted constant $C_{\nu ,\beta }:$

$$\begin{aligned} \int \limits _{b}^{\infty }\frac{M_{\nu +1/2}\left( c_{0}^{\prime }\left| s\right| \left( y+r_{\operatorname {R}}\right) \right) }{\left( \left| s\right| \left( y+r_{\operatorname {R}}\right) \right) ^{\nu +3/2}}dy&=C_{\nu ,\beta }\int \limits _{b}^{\infty }\left( \left| s\right| \left( y+r_{\operatorname {R}}\right) \right) ^{-\left( \nu +2\right) }dy\\&=C_{\nu ,\beta }\left| s\right| ^{-1}\left( \left| s\right| \left( b+r_{\operatorname {R}}\right) \right) ^{-\left( \nu +1\right) }\\&=C_{\nu ,\beta }\frac{1}{\left| s\right| \left( \max \left\{ 1,c_{0}^{\prime }\left| s\right| r_{\operatorname {R}}\right\} \right) ^{\nu +1}}. \end{aligned}$$

The integral over $\left( 0,b\right)$ is nonzero only for b>0, i.e. for $\left| s\right| r_{\operatorname {R}}<1/c_{0}^{\prime }$. We first consider the case $\nu \in \mathbb {Z}_{\ge 0}^{\operatorname {half}}$. Then, by an additional adjustment of the constant $C_{\nu ,\beta }$ it holds

$$\begin{aligned} \int \limits _{0}^{b}\frac{M_{\nu +1/2}\left( c_{0}^{\prime }\left| s\right| \left( y+r_{\operatorname {R}}\right) \right) }{\left( \left| s\right| \left( y+r_{\operatorname {R}}\right) \right) ^{\nu +3/2}}dy&=C_{\nu ,\beta }\int \limits _{0}^{\frac{1}{c_{0}^{\prime }\left| s\right| }-r_{\operatorname {R}}}\frac{1}{\left( \left| s\right| \left( y+r_{\operatorname {R}}\right) \right) ^{2\nu +2}}dy\\&\le C_{\nu ,\beta }\frac{1}{\left| s\right| }\frac{1}{\left( \left| s\right| r_{\operatorname {R}}\right) ^{2\nu +1}}. \end{aligned}$$

The estimate for the full integral is then given by:

$$ \int \limits _{0}^{\infty }\frac{M_{\nu +1/2}\left( c_{0}^{\prime }\left| s\right| \left( y+r_{\operatorname {R}}\right) \right) }{\left( \left| s\right| \left( y+r_{\operatorname {R}}\right) \right) ^{\nu +3/2}}dy\le C_{\nu ,\beta }\left\{ \begin{array}{lc} \frac{1}{\left| s\right| \left( \left| s\right| r_{\operatorname {R}}\right) ^{\nu +1}} &  {\text {for }}\left| s\right| r_{\operatorname {R}}\ge 1/c_{0}^{\prime },\\ \frac{1}{\left| s\right| \left( \left| s\right| r_{\operatorname {R}}\right) ^{2\nu +1}} &  {\text {for }}\left| s\right| r_{\operatorname {R}}<1/c_{0}^{\prime }. \end{array} \right. $$

It remains to consider the integral over $\left( 0,b\right)$ for ν=-1/2 and $\left| s\right| r_{\operatorname {R}}<1/c_{0}^{\prime }$. We get^6^

$$\begin{aligned} \int \limits _{0}^{b}\frac{M_{0}\left( c_{0}^{\prime }\left| s\right| \left( y+r_{\operatorname {R}}\right) \right) }{\left| s\right| \left( y+r_{\operatorname {R}}\right) }dy&\le C_{-1/2,\beta }c_{0}^{\prime } \int \limits _{0}^{\frac{1}{c_{0}^{\prime }\left| s\right| } -r_{\operatorname {R}}}\frac{\left( 1+\left| \ln \left( c_{0}^{\prime }\left| s\right| \left( y+r_{\operatorname {R}}\right) \right) \right| \right) }{c_{0}^{\prime }\left| s\right| \left( y+r_{\operatorname {R}}\right) }dy\\&=C_{-1/2,\beta }c_{0}^{\prime }\left( \frac{\left| \ln \left( c_{0}^{\prime }\left| s\right| r_{\operatorname {R}}\right) \right| }{|s|}+\frac{\ln ^{2}\left( c_{0}^{\prime }\left| s\right| r_{\operatorname {R}}\right) }{2|s|}\right) \\&\le C_{-1/2,\beta }c_{0}^{\prime }\frac{1+\ln ^{2}\left( c_{0}^{\prime }\left| s\right| r_{\operatorname {R}}\right) }{\left| s\right| }. \end{aligned}$$

This leads to

4.46 $$\begin{aligned} \left| \Theta _{\nu ,s}^{\operatorname {imp}}\left( \textbf{x} ,\textbf{y}\right) \right| \le C_{\nu ,\beta }\left\{ \begin{array}{ll} \left( \frac{\left| s\right| }{r_{\operatorname {R}}}\right) ^{\nu }\frac{1}{r_{\operatorname {R}}} &  {\text {for }}\left| s\right| r_{\operatorname {R}}\ge 1/c_{0}^{\prime },\\ \frac{1}{r_{\operatorname {R}}^{2\nu +1}} &  {\text {for }}\left| s\right| r_{\operatorname {R}}<1/c_{0}^{\prime }\wedge \nu \in \mathbb {Z}_{\ge 0}^{\operatorname {half}},\\ 1+\ln ^{2}\left( c_{0}^{\prime }\left| s\right| r_{\operatorname {R} }\right) &  {\text {for }}\left| s\right| r_{\operatorname {R}} <1/c_{0}^{\prime }\wedge \nu =-1/2 \end{array} \right. \end{aligned}$$

from which the assertion follows by some further adjustments of the constant $C_{\nu ,\beta }$. □

#### Remark 4.13

Let $\nu \in \mathbb {Z}_{\ge 0}^{\operatorname {half}}$, $\textbf{z} =\textbf{x}-\textbf{y}$, and $\textbf{z}_{-}=\textbf{x}-\textbf{Ry}$. Estimate (4.46) shows that the dominant singular behaviour of $\Theta _{\nu ,s}^{\operatorname {imp}}\left( \textbf{x},\textbf{y}\right)$ can be estimated for small $\left\| \textbf{z}_{-}\right\|$ by $O\left( \left\| \textbf{z}_{-}\right\| ^{-2\nu -1}\right)$. We emphasize that the product $\left( \Theta _{\nu ,s}^{\operatorname {refl}}\left( \textbf{x},\textbf{y}\right) +\Theta _{\nu ,s}^{\operatorname {imp}}\left( \textbf{x},\textbf{y}\right) \right) \left\| \textbf{z}_{-}\right\| ^{2\nu +1}$ (cf. (3.3)) is not necessarily smooth at $\left\| \textbf{z}_{-}\right\| =0$. Indeed, for ν=0 and β=1 the representation^7^ of $G_{\operatorname {half}}$

4.47 $$\begin{aligned} G_{\operatorname {half}}\left( \textbf{x},\textbf{y}\right) :=\frac{\operatorname {e}\nolimits ^{-s\left\| \textbf{z}\right\| }}{4\pi \left\| \textbf{z}\right\| }+\operatorname {e} \nolimits ^{-s\left\| \textbf{z}_{-}\right\| }\left( \frac{1}{4\pi \left\| \textbf{z}_{-}\right\| }-\frac{s}{2\pi }U\left( 1,1,s\left( \left\| \textbf{z}_{-}\right\| +\left( z_{-}\right) _{3}\right) \right) \right) \end{aligned}$$

with Tricomi’s (confluent hypergeometric) function $U\left( a,b,z\right)$ (see [14, 13.2.6]) is derived in [23, Sec. 4]. This leads to

4.48 $$\begin{aligned} \left( \Theta _{0,s}^{\operatorname {refl}}\left( \textbf{x},\textbf{y} \right) +\Theta _{0,s}^{\operatorname {imp}}\left( \textbf{x},\textbf{y} \right) \right) \left\| \textbf{z}_{-}\right\| =\frac{1}{4\pi } -\frac{s}{2\pi }\left\| \textbf{z}_{-}\right\| U\left( 1,1,s\left( \left\| \textbf{z}_{-}\right\| +\left( z_{-}\right) _{3}\right) \right) . \end{aligned}$$

Clearly, the dominant singular behaviour of the first two summands in (4.47) for small arguments is $\left\| \textbf{z}\right\| ^{-1}$ for the first and $\left\| \textbf{z}_{-}\right\| ^{-1}$ for the second summand which shows that (4.46) is sharp in this case. However, the dominant singular behaviour of Tricomi’s function is logarithmic: $U\left( 1,1,\rho \right) =-\left( \gamma +1+\log \rho \right) +o\left( 1\right)$ as $\rho \rightarrow 0$ (see [29, Chap. 48:9]) and the third term in (4.47) has a singularity of lower order compared to the second summand. In this way, the right-hand side in (4.48) is not analytic.

## Estimate of $\tilde{\mu }$

The estimate of $\tilde{\mu }$ employs the representation introduced in Remark 4.9, and we recall the notation

$$ \chi =a^{2}-\left( 1-\beta ^{2}\right) \omega ^{2}\quad {\text {with}}\quad a:=y+r_{+},\quad \omega ^{2}:=\left\langle \textbf{z}^{\prime },\textbf{z} ^{\prime }\right\rangle . $$

### Proof of Lemma 4.10

First, estimates for χ will be derived. An explicit computation yields

5.1 $$\begin{aligned} \chi&=\left( y+r+\beta z_{d}\right) ^{2}-\left( 1-\beta ^{2}\right) \left( r^{2}-z_{d}^{2}\right) \nonumber \\&=y^{2}+z_{d}^{2}+\beta ^{2}r^{2}+2yr+2\beta yz_{d}+2\beta rz_{d}. \end{aligned}$$

For the real part, we obtain

$$ \operatorname {Re}\chi =y^{2}+\operatorname {Re}\left( z_{d}^{2}\right) +\beta ^{2}\operatorname {Re}\left( r^{2}\right) +2y\operatorname {Re}r+2\beta y\operatorname {Re}z_{d}+2\beta \operatorname {Re}\left( rz_{d}\right) . $$

Let $\left( \textbf{x},\textbf{y}\right) \in \overrightarrow{\mathcal {E} }_{\textbf{a},\textbf{b},\textbf{c},\textbf{d}}\left( \kappa \right)$, $\textbf{z}:=\mathbf {x-Ry}$, and $r_{\operatorname {R}}:=\left\| \textbf{z}\right\|$. As a first condition we impose $C_{\mathcal {E}}>3$; then a straightforward calculation shows that $\frac{\beta ^{2}}{C_{\mathcal {E} }\left( 1+\beta \right) ^{3}}<\min \left\{ 1/6,1/\left( 3\beta \right) \right\}$ and all estimates in Lemmata 4.5 and 4.7 are applicable. This implies the existence^8^ of some $C_{1}\ge 1$ such that

5.2 $$\begin{aligned} \begin{array}{ll} \left| \operatorname {Im}r\right| &  \overset{\text {(4.17)} }{\le }C_{1}\kappa r_{\operatorname {R}},\\ \left| \operatorname {Im}z_{d}\right| &  \overset{\text {(4.25)}}{\le }C_{1}\kappa r_{\operatorname {R}},\\ \operatorname {Re}r &  \overset{\text {(4.16)}}{\le }C_{1} r_{\operatorname {R}},\\ \operatorname {Re}r &  \overset{\text {(4.16)}}{\ge }C_{1}^{-1} r_{\operatorname {R}},\\ \operatorname {Re}\left( r^{2}\right) &  \overset{\text {(4.27a)}}{\ge }\left( C_{1}^{-1}r_{\operatorname {R}}\right) ^{2},\\ \operatorname {Re}z_{d} &  \overset{\text {(4.26)}}{\le } C_{1}r_{\operatorname {R}},\\ \operatorname {Re}z_{d} &  =\xi _{d}+y_{d}+\operatorname {Re}x_{d}-\xi _{d}\overset{\xi _{d}+y_{d}>0}{>}-\left| x_{d}-\xi _{d}\right| \overset{\text {(4.23)}}{\ge }-C_{1}\kappa r_{\operatorname {R}},\\ \operatorname {Re}\left( rz_{d}\right) &  =\operatorname {Re}r\operatorname {Re} z_{d}-\operatorname {Im}r\operatorname {Im}z_{d}\ge -C_{1}^{2}\kappa r_{\operatorname {R}}^{2},\\ \left| \operatorname {Im}\left( rz_{d}\right) \right| &  \le \left| \operatorname {Im}r\right| \left| \operatorname {Re} z_{d}\right| +\left| \operatorname {Re}r\right| \left| \operatorname {Im}z_{d}\right| \le C_{1}^{2}\kappa r_{\operatorname {R} }^{2}. \end{array} \end{aligned}$$

In this way, we obtain

5.3 $$\begin{aligned} \operatorname {Re}\chi&\ge y^{2}-\left( C_{1}\kappa r_{\operatorname {R} }\right) ^{2}+\beta ^{2}\left( C_{1}^{-1}r_{\operatorname {R}}\right) ^{2}+2C_{1}^{-1}yr_{\operatorname {R}}+2\beta y\left( -C_{1}\kappa r_{\operatorname {R}}\right) +2\beta \left( -C_{1}^{2}\kappa r_{\operatorname {R}}^{2}\right) \nonumber \\&\ge y^{2}+\left( \left( \frac{\beta }{C_{1}}\right) ^{2}-C_{1}^{2} \kappa \left( 2\beta +\kappa \right) \right) r_{\operatorname {R}} ^{2}+2yr_{\operatorname {R}}\left( C_{1}^{-1}-\beta C_{1}\kappa \right) . \end{aligned}$$

For the prefactors, it holds for the considered range of κ:

5.4 $$\begin{aligned} \left( \frac{\beta }{C_{1}}\right) ^{2}-C_{1}^{2}\kappa \left( 2\beta +\kappa \right)&\ge \beta ^{2}\left( C_{1}^{-2}-C_{1}^{2}\frac{\beta }{C_{\mathcal {E}}\left( 1+\beta \right) ^{3}}\left( 2+\frac{\beta }{C_{\mathcal {E}}\left( 1+\beta \right) ^{3}}\right) \right) \nonumber \\&\ge \beta ^{2}\left( C_{1}^{-2}-\frac{C_{1}^{2}}{C_{\mathcal {E}}}\left( 2+\frac{1}{C_{\mathcal {E}}}\right) \right) , \end{aligned}$$

5.5 $$\begin{aligned} C_{1}^{-1}-\beta C_{1}\kappa&\ge C_{1}^{-1}-\frac{C_{1}}{C_{\mathcal {E}}}. \end{aligned}$$

A possible adjustment of $C_{\mathcal {E}}$ (only depending on the number $C_{1}>0$) implies that the right-hand side in (5.5) is positive (so that the last term in (5.3) can be dropped in a lower bound) and there is a number $0<C_{2}\le 1$ such that the right-hand side in (5.4) is bounded from below by $C_{2}\beta ^{2}$. In this way

5.6 $$\begin{aligned} \operatorname {Re}\chi \ge y^{2}+C_{2}\beta ^{2}r_{\operatorname {R}}^{2} \end{aligned}$$

follows.

For the imaginary part, we start from (5.1) and obtain

$$ \operatorname {Im}\chi =2\operatorname {Re}z_{d}\operatorname {Im}z_{d}+2\beta ^{2}\operatorname {Re}r\operatorname {Im}r+2y\operatorname {Im}r+2\beta y\operatorname {Im}z_{d}+2\beta \operatorname {Im}\left( rz_{d}\right) . $$

This implies the estimate of the modulus

$$\begin{aligned} \left| \operatorname {Im}\chi \right|&\le 2C_{1}^{2}\kappa r_{\operatorname {R}}^{2}+2\beta ^{2}C_{1}^{2}\kappa r_{\operatorname {R}} ^{2}+2C_{1}\kappa yr_{\operatorname {R}}+2C_{1}\beta \kappa yr_{\operatorname {R}}+2C_{1}^{2}\beta \kappa r_{\operatorname {R}}^{2}\\&\le \kappa \left( 2yC_{1}\left( 1+\beta \right) r_{\operatorname {R} }+2C_{1}^{2}\left( 1+\beta \right) ^{2}r_{\operatorname {R}}^{2}\right) \\&\le \kappa \left( y^{2}+3\left( 1+\beta \right) ^{2}C_{1}^{2} r_{\operatorname {R}}^{2}\right) . \end{aligned}$$

The ratio of the imaginary and real part of χ can be bounded by

5.7 $$\begin{aligned} \frac{\left| \operatorname {Im}\chi \right| }{\operatorname {Re}\chi } \le \frac{y^{2}+3\left( 1+\beta \right) ^{2}C_{1}^{2}r_{\operatorname {R} }^{2}}{y^{2}+C_{2}\beta ^{2}r_{\operatorname {R}}^{2}}\kappa \le 3\frac{C_{1}^{2}}{C_{2}}\frac{\left( 1+\beta \right) ^{2}}{\beta ^{2}}\kappa . \end{aligned}$$

For the modulus, it holds

$$\begin{aligned} \left| \chi \right|&\le \left| y+r_{+}\right| ^{2}+\left( 1+\beta ^{2}\right) r_{\operatorname {R}}^{2}\le 2y^{2}+2\left| r_{+}\right| ^{2}+\left( 1+\beta ^{2}\right) r_{\operatorname {R}}^{2}\\&\overset{\text {(4.30), (4.16)}}{\le } 2y^{2}+3\left( 1+\beta \right) ^{2}r_{\operatorname {R}}^{2}. \end{aligned}$$

We have collected all ingredients for deriving estimates for $\sqrt{\chi }$. For the modulus, we obtain

5.8 $$\begin{aligned} \left| \sqrt{\chi }\right| \overset{\text {(4.11)}}{=} \sqrt{\left| \chi \right| }\le \sqrt{3}\left( y+\left( 1+\beta \right) r_{\operatorname {R}}\right) \end{aligned}$$

and for the real part

5.9 $$\begin{aligned} \operatorname {Re}\sqrt{\chi }&\overset{\text {(4.12)}}{\ge }\sqrt{\left| \chi \right| }\left( 1-\frac{1}{8}\left( \frac{\operatorname {Im}\chi }{\operatorname {Re}\chi }\right) ^{2}\right) \ge \sqrt{\operatorname {Re}\chi }\left( 1-\frac{1}{8}\left( \frac{\operatorname {Im}\chi }{\operatorname {Re}\chi }\right) ^{2}\right) \nonumber \\&\overset{\text {(5.6), (5.7)}}{\ge }\left( 1-\frac{1}{8}\left( 3\frac{C_{1}^{2}}{C_{2}}\frac{\left( 1+\beta \right) ^{2}}{\beta ^{2}}\kappa \right) ^{2}\right) \sqrt{y^{2}+C_{2}\beta ^{2} r_{\operatorname {R}}^{2}}. \end{aligned}$$

For the considered range of κ, it holds

$$ \frac{1}{8}\left( 3\frac{C_{1}^{2}}{C_{2}}\frac{\left( 1+\beta \right) ^{2} }{\beta ^{2}}\kappa \right) ^{2}\le \frac{9}{8}\frac{C_{1}^{4}}{C_{\mathcal {E} }^{2}C_{2}^{2}}. $$

Again, by a possible adjustment of $C_{\mathcal {E}}$ only depending on the numbers $C_{1}$, $C_{2}$, the prefactor in (5.9) is bounded from below by 1/2 and

5.10 $$\begin{aligned} \operatorname {Re}\sqrt{\chi }\ge \frac{1}{2}\sqrt{y^{2}+C_{2}\beta ^{2}r_{\operatorname {R}}^{2}}. \end{aligned}$$

Using the definitions in (4.41), it is a straightforward calculation to verify $t+\beta \tilde{\mu }=\sqrt{\chi }$ and (4.42c) follows.

Next the modulus $\left| \tilde{\mu }\left( \textbf{z},y\right) \right|$ will be estimated and the representation of $\tilde{\mu }$ in (4.41) is employed. For the numerator, we get

$$\begin{aligned} \left| a\sqrt{\chi }+\beta \omega ^{2}\right|&\overset{\text {(5.8)}}{\le }\sqrt{3}\left( y+\left| r_{+}\right| \right) \left( y+\left( 1+\beta \right) r_{\operatorname {R}}\right) +\beta r_{\operatorname {R}}^{2}\\&\overset{\text {(4.30), (4.16)}}{\le }\sqrt{3}\left( y+\left( 1+\beta \right) r_{\operatorname {R}}\right) ^{2}+\beta r_{\operatorname {R}}^{2}\\&\le 3\left( y+\left( 1+\beta \right) r_{\operatorname {R}}\right) ^{2} \end{aligned}$$

and for the denominator:

$$\begin{aligned} \left| \beta a+\sqrt{\chi }\right|&\ge \beta \left( y+\operatorname {Re}r_{+}\right) +\operatorname {Re}\sqrt{\chi }\\&\overset{\text {(5.2), (5.10)}}{\ge } \beta \left( y+\frac{1-\beta C_{1}^{2}\kappa }{C_{1}}r_{\operatorname {R} }\right) +\frac{1}{2}\sqrt{y^{2}+C_{2}\beta ^{2}r_{\operatorname {R}}^{2}}. \end{aligned}$$

The bound on κ implies $C_{1}\beta \kappa \le C_{1}/C_{\mathcal {E}}$ and for sufficiently large $C_{\mathcal {E}}$ (only depending on $C_{1}$) it holds $1-\beta C_{1}^{2}\kappa \ge 1/2$ so that

5.11 $$\begin{aligned} \operatorname {Re}r_{+}\overset{\text {(5.2)}}{\ge }\left( C_{1}^{-1}-C_{1}\beta \kappa \right) r_{\operatorname {R}}\ge \frac{r_{\operatorname {R}}}{2C_{1}}. \end{aligned}$$

Thus,

5.12 $$\begin{aligned} \left| \beta a+\sqrt{\chi }\right|&\ge \beta \left( y+\frac{r_{\operatorname {R}}}{2C_{1}}\right) +\frac{1}{2\sqrt{2}}\left( y+\sqrt{C_{2}}\beta r_{\operatorname {R}}\right) \nonumber \\&\ge \left( \frac{1}{2\sqrt{2}}+\beta \right) y+\beta \left( \frac{\sqrt{C_{2}}}{2\sqrt{2}}+\frac{1}{2C_{1}}\right) r_{\operatorname {R} }\nonumber \\&\ge C_{3}\left( \left( 1+\beta \right) y+\beta r_{\operatorname {R} }\right) \end{aligned}$$

for some $C_{3}$ depending only on $C_{1}$ and $C_{2}$. This leads to the upper estimate (cf. (4.41))

$$ \left| \tilde{\mu }\left( \textbf{z},y\right) \right| \le \frac{3}{C_{3}\left( 1+\beta \right) }\frac{y+\left( 1+\beta \right) r_{\operatorname {R}}}{y+\frac{\beta }{\left( 1+\beta \right) } r_{\operatorname {R}}}\left( y+\left( 1+\beta \right) r_{\operatorname {R} }\right) \le \frac{3}{C_{3}}\frac{1+\beta }{\beta }\left( y+\left( 1+\beta \right) r_{\operatorname {R}}\right) . $$

To derive a lower estimate for the real part, we use

5.13 $$\begin{aligned} \operatorname {Re}\tilde{\mu }\left( \textbf{z},y\right) =\frac{\operatorname {Re}\left( \left( a\sqrt{\chi }+\beta \omega ^{2}\right) \left( \overline{\beta a+\sqrt{\chi }}\right) \right) }{\left| \beta a+\sqrt{\chi }\right| ^{2}}=\frac{N}{\left| \beta a+\sqrt{\chi }\right| ^{2}} \end{aligned}$$

with

$$ N:=\beta \left| a\right| ^{2}\operatorname {Re}\sqrt{\chi }+\left| \chi \right| \operatorname {Re}a+\beta ^{2}\operatorname {Re}\left( a\omega ^{2}\right) +\beta \operatorname {Re}\left( \omega ^{2}\overline{\sqrt{\chi }}\right) $$

and estimate the terms in *N* step by step. From (5.2) one concludes that

5.14 $$\begin{aligned} \left| a\right| ^{2}&\ge \left( y+\operatorname {Re}r_{+}\right) ^{2}\overset{\text {(5.11)}}{\ge }\left( y+\frac{r_{\operatorname {R}}}{2C_{1}}\right) ^{2},\nonumber \\ \operatorname {Re}\sqrt{\chi }&\overset{\text {(5.10)}}{\ge }\frac{1}{2}\sqrt{y^{2}+C_{2}\beta ^{2}r_{\operatorname {R}}^{2}} \ge \frac{1}{3}\left( y+\sqrt{C_{2}}\beta r_{\operatorname {R}}\right) , \end{aligned}$$

5.15 $$\begin{aligned} \left| \chi \right|&\ge \operatorname {Re}\chi \overset{\text {(5.6)}}{\ge }y^{2}+C_{2}\beta ^{2}r_{\operatorname {R} }^{2},\nonumber \\ \operatorname {Re}a&\ge y+\frac{r_{\operatorname {R}}}{2C_{1} }. \end{aligned}$$

This allows us to estimate the first two summands in the definition of *N*

$$\begin{aligned} \beta \left| a\right| ^{2}\operatorname {Re}\sqrt{\chi }+\left| \chi \right| \operatorname {Re}a&\ge \left( y+\frac{r_{\operatorname {R} }}{2C_{1}}\right) \left( \frac{\beta }{3}\left( y+\frac{r_{\operatorname {R} }}{2C_{1}}\right) \left( y+\sqrt{C_{2}}\beta r_{\operatorname {R}}\right) +\left( y^{2}+C_{2}\beta ^{2}r_{\operatorname {R}}^{2}\right) \right) \\&\ge \left( y+\frac{r_{\operatorname {R}}}{2C_{1}}\right) \left( \frac{\beta }{3}\left( y^{2}+\frac{\sqrt{C_{2}}}{2C_{1}}\beta r_{\operatorname {R}}^{2}\right) +y^{2}+C_{2}\beta ^{2}r_{\operatorname {R} }^{2}\right) \\&\ge \left( y+\frac{r_{\operatorname {R}}}{2C_{1}}\right) \left( \left( 1+\frac{\beta }{3}\right) y^{2}+\left( \frac{\sqrt{C_{2}}}{6C_{1}} +C_{2}\right) \beta ^{2}r_{\operatorname {R}}^{2}\right) \\&\ge C_{4}\left( y+\frac{r_{\operatorname {R}}}{2C_{1}}\right) \left( \left( 1+\beta \right) y^{2}+\beta ^{2}r_{\operatorname {R}}^{2}\right) \end{aligned}$$

for some $C_{4}$ depending only on $C_{1}$ and $C_{2}$. To estimate the last two summands in *N*, we start with the relations

$$\begin{aligned} \operatorname {Re}\left( a\omega ^{2}\right)&=\operatorname {Re} a\operatorname {Re}\left( \omega ^{2}\right) -\operatorname {Im} a\operatorname {Im}\left( \omega ^{2}\right) ,\\ \operatorname {Re}\left( \omega ^{2}\overline{\sqrt{\chi }}\right)&=\operatorname {Re}\left( \omega ^{2}\right) \operatorname {Re}\sqrt{\chi }+\operatorname {Im}\sqrt{\chi }\operatorname {Im}\left( \omega ^{2}\right) . \end{aligned}$$

For the single factors, we employ (4.27) and

$$\begin{aligned} \operatorname {Re}\left( z_{d}^{2}\right)&=\left( \operatorname {Re} z_{d}\right) ^{2}-\left( \operatorname {Im}z_{d}\right) ^{2} \overset{\text {(4.26)}}{\le }r_{\operatorname {R}}^{2},\\ \left| \operatorname {Re}a\right|&\le y+\left| \operatorname {Re}\left( r+\beta z_{d}\right) \right| \overset{\text {(4.16), (4.26)}}{\le }y+\left( 1+\beta \right) r_{\operatorname {R}},\\ \left| \operatorname {Im}a\right|&\le \operatorname {Im}\left( r+\beta z_{d}\right) \overset{\text {(4.17), (4.25)}}{\le }2\kappa \left( 2+\beta \right) r_{\operatorname {R}} \end{aligned}$$

to get

$$ \operatorname {Re}\left( \omega ^{2}\right) \ge \operatorname {Re}\left\langle \textbf{z},\textbf{z}\right\rangle -\operatorname {Re}\left( z_{d}^{2}\right) \ge \left( 1-8\kappa ^{2}\right) r_{\operatorname {R}}^{2} -r_{\operatorname {R}}^{2}\ge -8\kappa ^{2}r_{\operatorname {R}}^{2} \quad {\text {and}}\quad \left| \operatorname {Im}\left( \omega ^{2}\right) \right| \le 4\kappa r_{\operatorname {R}}^{2}, $$

while the estimate of $\sqrt{\chi }$ follows in a similar fashion as (5.9) from (4.13)

$$ \left| \operatorname {Im}\sqrt{\chi }\right| \le \frac{\left| \operatorname {Im}\chi \right| }{2\operatorname {Re}\chi }\sqrt{\left| \chi \right| }\overset{\text {(5.7), (5.8)}}{\le }\frac{3\sqrt{3}C_{1}^{2}}{2C_{2}}\frac{\left( 1+\beta \right) ^{2}}{\beta ^{2}}\kappa \left( y+\left( 1+\beta \right) r_{\operatorname {R} }\right) . $$

In this way, we obtain for the real parts of the products:

$$\begin{aligned} \operatorname {Re}\left( a\omega ^{2}\right)&\overset{\text {(4.30), (5.2)}}{\ge }-C_{5}\left( y+\left( 1+\beta \right) r_{\operatorname {R}}\right) \kappa ^{2} r_{\operatorname {R}}^{2},\\ \operatorname {Re}\left( \omega ^{2}\overline{\sqrt{\chi }}\right)&\overset{\text {(5.8)}}{\ge }-C_{5}\frac{\left( 1+\beta \right) ^{2}}{\beta ^{2}}\left( y+\left( 1+\beta \right) r_{\operatorname {R}}\right) \kappa ^{2}r_{\operatorname {R}}^{2} \end{aligned}$$

for a constant $C_{5}$ which only depends on the numbers $C_{1}$ and $C_{2}$. The sum of both terms can be estimated by

$$\begin{aligned} \operatorname {Re}\left( a\omega ^{2}\right) +\operatorname {Re}\left( \omega ^{2}\overline{\sqrt{\chi }}\right)&\ge -2C_{5}\frac{\left( 1+\beta \right) ^{2}}{\beta ^{2}}\left( y+\left( 1+\beta \right) r_{\operatorname {R}}\right) \kappa ^{2}r_{\operatorname {R}}^{2}\\&\ge -\frac{2C_{5}}{C_{\mathcal {E}}^{2}}\frac{\beta ^{2}}{\left( 1+\beta \right) ^{4}}\left( y+\left( 1+\beta \right) r_{\operatorname {R} }\right) r_{\operatorname {R}}^{2}. \end{aligned}$$

Thus, for the numerator *N* in (5.13) it follows

$$\begin{aligned} N&\ge C_{4}\left( y+\frac{r_{\operatorname {R}}}{2C_{1}}\right) \left( \left( 1+\beta \right) y^{2}+\beta ^{2}r_{\operatorname {R}}^{2}\right) -\frac{2C_{5}}{C_{\mathcal {E}}^{2}}\frac{1}{\left( 1+\beta \right) ^{3} }\left( y+r_{\operatorname {R}}\right) \beta ^{2}r_{\operatorname {R}}^{2}\\&\ge \left( C_{4}\left( y+\frac{r_{\operatorname {R}}}{2C_{1}}\right) -\frac{2C_{5}}{C_{\mathcal {E}}}\left( y+\frac{r_{\operatorname {R}} }{C_{\mathcal {E}}}\right) \right) \left( \left( 1+\beta \right) y^{2}+\beta ^{2}r_{\operatorname {R}}^{2}\right) . \end{aligned}$$

Again, by a possible adjustment of $C_{\mathcal {E}}$ only depending on the numbers $C_{1}$, $C_{2}$, $C_{4}$, $C_{5}$ we end up with

$$ N\ge C_{6}\left( y+r_{\operatorname {R}}\right) \left( \left( 1+\beta \right) y^{2}+\beta ^{2}r_{\operatorname {R}}^{2}\right) . $$

For the denominator, it holds

5.16 $$\begin{aligned} \left| \beta a+\sqrt{\chi }\right| \le \beta \left( y+\left| r_{+}\right| \right) +\left| \sqrt{\chi }\right| \overset{\text {(4.33), (5.8)}}{\le }\left( \beta +\sqrt{3}\right) \left( y+\left( 1+\beta \right) r_{\operatorname {R} }\right) . \end{aligned}$$

The combination of these two inequalities leads to:

$$\begin{aligned} \operatorname {Re}\tilde{\mu }\left( \textbf{z},y\right)&\ge \frac{C_{6}\left( y+r_{\operatorname {R}}\right) \left( \left( 1+\beta \right) y^{2}+\beta ^{2}r_{\operatorname {R}}^{2}\right) }{\left( \beta +\sqrt{3}\right) ^{2}\left( y+\left( 1+\beta \right) r_{\operatorname {R} }\right) ^{2}}\ge \frac{\left( 1+\beta \right) C_{6}}{2\left( \beta +\sqrt{3}\right) ^{2}}\left( \frac{y+\frac{\beta }{\sqrt{1+\beta } }r_{\operatorname {R}}}{y+\left( 1+\beta \right) r_{\operatorname {R}} }\right) ^{2}\left( y+r_{\operatorname {R}}\right) \\&\ge \frac{C_{6}}{2\left( \beta +\sqrt{3}\right) ^{2}}\frac{\beta ^{2} }{\left( 1+\beta \right) ^{2}}\left( y+r_{\operatorname {R}}\right) \end{aligned}$$

and the assertion for $\operatorname {Re}\tilde{\mu }$ follows.

Next, we estimate the modulus of $\tilde{\mu }^{\prime }$ and recall (2.10):

5.17 $$\begin{aligned} \tilde{\mu }^{\prime }\left( \textbf{z},y\right)&=\frac{\partial \tilde{\mu }\left( \textbf{z},y\right) }{\partial y}=\frac{t\left( \textbf{z} ,y\right) }{t\left( \textbf{z},y\right) +\beta \tilde{\mu }\left( \textbf{z},y\right) }\nonumber \\&\overset{\text {(2.10), (4.41)}}{=}\frac{a^{2} -\omega ^{2}}{a\left( a+\beta \sqrt{\chi }\right) +\left( \beta ^{2}-1\right) \omega ^{2}}=\frac{a^{2}-\omega ^{2}}{\sqrt{\chi }\left( \beta a+\sqrt{\chi }\right) }. \end{aligned}$$

For the numerator, we get

$$ \left| a^{2}-\omega ^{2}\right| \le \left| a\right| ^{2}+\left| \omega \right| ^{2}\overset{\text {(4.33)}}{\le }\left( y+\left( 1+\beta \right) r_{\operatorname {R}}\right) ^{2}+r_{\operatorname {R}}^{2}\le 2\left( y+\left( 1+\beta \right) r_{\operatorname {R}}\right) ^{2} $$

and for the denominator:

$$ \left| \sqrt{\chi }\right| \left| \beta a+\sqrt{\chi }\right| \overset{\text {(5.14),(5.12)}}{\ge }\frac{C_{3}}{3}\left( y+\sqrt{C_{2}}\beta r_{\operatorname {R}}\right) \left( \left( 1+\beta \right) y+\beta r_{\operatorname {R}}\right) . $$

This leads to

$$\begin{aligned} \left| \tilde{\mu }^{\prime }\left( \textbf{z},y\right) \right|&\le \frac{6}{C_{3}}\frac{\left( y+\left( 1+\beta \right) r_{\operatorname {R}}\right) ^{2}}{\left( y+\sqrt{C_{2}}\beta r_{\operatorname {R}}\right) \left( \left( 1+\beta \right) y+\beta r_{\operatorname {R}}\right) }\le \frac{6}{C_{3}}\frac{1+\beta }{\beta } \frac{y+\left( 1+\beta \right) r_{\operatorname {R}}}{y+\sqrt{C_{2}}\beta r_{\operatorname {R}}}\\&\le \frac{6}{C_{3}}\frac{1+\beta }{\beta }\max \left\{ 1,\frac{1+\beta }{\sqrt{C_{2}}\beta }\right\} \end{aligned}$$

and finally to the assertion □

## Data Availability

There are no data attached to this paper.

## Footnotes

1 Note that the domain of the function $\Theta _{\nu ,s}^{\operatorname {illu}}$ in (3.3) is $H_{+}\times H_{+}$. However, for the analysis of $\Theta _{\nu ,s}^{\operatorname {refl}}$ it turns out to be useful to consider $\Theta _{\nu }^{\operatorname {illu}}$ on the larger domain $\mathbb {R} ^{d}\times \mathbb {R}^{d}$.

2 Tensor notation: $\hat{L}_{ {\boldsymbol{\mu }},m}\left( \textbf{x}\right) :=\left( {\displaystyle \bigotimes \limits _{\ell =1}^{d}} \hat{L}_{\mu _{\ell },m}\right) \left( \textbf{x}\right) := {\displaystyle \prod \limits _{\ell =1}^{d}} \hat{L}_{\mu _{\ell },m}\left( x_{\ell }\right)$ and $\left( L_{ \boldsymbol{\mu },m}^{\left[ \textbf{a},\textbf{b}\right] }\otimes L_{ \boldsymbol{\nu },m}^{\left[ \textbf{c},\textbf{d}\right] }\right) \left( \textbf{x},\textbf{y}\right) :=L_{ {\boldsymbol{\mu }},m}^{\left[ \textbf{a},\textbf{b}\right] }\left( \textbf{x}\right) L_{ \boldsymbol{\nu },m}^{\left[ \textbf{c},\textbf{d}\right] }\left( \textbf{y}\right)$.

3 Here and the following, an *analytic extension* of a function *k* to $\overrightarrow{\mathcal {E} }_{\textbf{a},\textbf{b},\textbf{c},\textbf{d}}\left( \boldsymbol{\rho } _{1},\boldsymbol{\rho } _{2}\right)$ means that the extended function *k* is continuous on $\overrightarrow{\mathcal {E}}_{\textbf{a},\textbf{b},\textbf{c},\textbf{d} }\left( \boldsymbol{\rho } _{1},\boldsymbol{\rho } _{2}\right)$ and for each fixed *j* and fixed ${\textbf{x}}\in [\textbf{a},\textbf{b}]$, ${\textbf{y}}\in [\textbf{c},\textbf{d}]$ the univariate functions $z\mapsto k(\left( x_{1},\ldots ,x_{j-1},z,x_{j+1},\ldots ,x_{d}\right) ,{\textbf{y}})$ and $z\mapsto k({\textbf{x} },\left( y_{1},\ldots ,y_{j-1},z,y_{j+1},\ldots ,y_{d}\right) )$ are holomorphic on the open sets $\operatorname {int}{\mathcal {E}}_{a_{j},b_{j} }^{(\rho _{1})_{j}}$ and $\operatorname {int}{\mathcal {E}}_{c_{j},d_{j}} ^{(\rho _{2})_{j}}$, respectively.

4 Here, rather generously, the maximal prefactor $2\chi \left( 1\right) =\pi$ for the estimate of the variational operator in [14, 10.40.12] has been used.

5 Here the property $\arctan x\le x$ for x≥0 is used.

6 It holds

$$ \int \limits _{0}^{\frac{1}{c_{0}^{\prime }\left| s\right| } -r_{\operatorname {R}}}\frac{\left| \ln \left( c_{0}^{\prime }\left| s\right| \left( y+r_{\operatorname {R}}\right) \right) \right| }{c_{0}^{\prime }\left| s\right| \left( y+r_{\operatorname {R} }\right) }dy=\frac{\ln ^{2}\left( c_{0}^{\prime }\left| s\right| r_{\operatorname {R}}\right) }{2c_{0}^{\prime }|s|}. $$

7 A similar representation can be found for different impedance parameter β in [21, (5.105)]

8 We omit a sharper specification of the involved constants for the sake of readability.
